# Smart tools and orthogonal click-like reactions onto small unilamellar vesicles: Additional molecular data

**DOI:** 10.1016/j.dib.2015.08.014

**Published:** 2015-08-28

**Authors:** Maria Vittoria Spanedda, Christophe Salomé, Benoît Hilbold, Etienne Berner, Béatrice Heurtault, Sylvie Fournel, Benoît Frisch, Line Bourel-Bonnet

**Affiliations:** Laboratoire de Conception et Application de Molécules Bioactives, Equipe de BioVectorologie, UMR 7199 – CNRS/Université de Strasbourg, Faculté de Pharmacie, 74 route du Rhin, BP 60024, 67401 Illkirch Cedex, France

## Abstract

We present here the synthetic routes and the experimental data (NMR and MS spectra) for model reactions for copper-free Huisgen 1,4-cycloaddition, Staudinger ligation and for addition of a dithiol on a dibromomaleimide ring. Starting materials were synthesized from the commercially available 4-chlorophenethylamine, previously described 2-(cyclooct-2-yn-1-yloxy)acetic acid, 1-fluorocyclooct-2-ynecarboxylic acid, commercial 2-(diphenylphosphino)terephthalic acid 1-methyl 4-pentafluorophenyl diester and dibromomaleimide. In all cases, the expected compounds were obtained with good yield (50% to quantitative). A novel synthesis of the lipid anchor DOGP_3_NH_2_ is also described. These data were used as basis for the study reported in the article “Smart Tools and Orthogonal Click-like Reactions onto Small Unilamellar Vesicles” in Chemistry and Physics of Lipids [1].

**Table:** Specifications table.

Subject area	Chemistry
More specific subject area	Bioconjugation
Type of data	Experimental synthesis protocols, analysis description, NMR and MS spectra
How data was acquired	^1^H NMR spectra at either 300 MHz, 400 MHz or 500 MHz and ^13^C NMR spectra at either 75 MHz, 100 MHz or 133 MHz recorded on Brucker spectrometers either 300, 400 or 500 respectively with residual undeuterated solvent as internal reference. High-resolution mass spectra (HRMS) obtained using an Agilent Q-TOF (time of flight) 6520 and low-resolution mass spectra (LRMS) using an Agilent MSD 1200 SL (ESI/APCI). Analytical RP–HPLC–MS performed using a C18 column (30 mm×1 mm; 1.9 μm) using the following parameters: (1) the eluent system A (0.05% TFA in H_2_O) and B (0.05% TFA in acetonitrile); (2) the linear gradient *t*=0 min with 98% A, *t*=5 min with 5% A, *t*=6 min with 5% A, *t*=7 min with 98% A, and *t*=9 min with 98% A; (3) flow rate of 0.3 mL min^-1^; (4) column temperature 50 °C; (5) ratio of products determined by integration of spectra recorded at 210 or 254 nm; and (6) ionization mode ESI.
Data format	Analyzed data
Experimental factors	Starting compounds were either purchased or synthesized using already published synthetic protocols
Experimental features	Compounds were synthesized and their structure was identified by NMR and confirmed by mass spectrometry
Data source location	Illkirch, France
Data accessibility	Data are provided in the paper

## Value of the data

• The data presented prove the efficiency of bioconjugation reactions and allow reproducibility of the syntheses.
• The overall work provides further tools for surface modification of liposomes.
• The article describes a novel, easier synthesis of the lipid anchor DOGP_3_NH_2_.

## 1 Data

All the described ^1^H NMR and ^13^C NMR spectra, as well as MS spectra, are available as annexes to this article.

^1^H NMR spectra at either 300 MHz, 400 MHz or 500 MHz and ^13^C NMR spectra at either 75 MHz, 100 MHz or 133 MHz were recorded on Brucker spectrometers either 300, 400 or 500 respectively with residual undeuterated solvent as internal reference. All chemical shift values (*δ*), coupling constants (*J*) and the multiplicity (s=singlet, d=doublet, t=triplet, m=multiplet, br=broad) are quoted in ppm and in Hz, respectively. High-resolution mass spectra (HRMS) were obtained using an Agilent Q-TOF (time of flight) 6520 and low-resolution mass spectra (LRMS) using an Agilent MSD 1200 SL (ESI/APCI).

## 2 Experimental design, materials and methods

Reagent grade solvents were used without further purification. Polymer supported triphenylphosphine and anhydrous CH_2_Cl_2_ were purchased from Sigma-Aldrich. The PyBOP was purchased from Novabiochem, the DIEA was from Alfa Aesar and both were used without further purification. Column chromatography was carried out on silica gel 60 (Merck, 70−230 mesh). Analytical RP–HPLC–MS was performed using a C18 column (30 mm×1 mm; 1.9 μm) using the following parameters: (1) the eluent system A (0.05% TFA in H_2_O) and B (0.05% TFA in acetonitrile); (2) the linear gradient *t*=0 min with 98% A, *t*=5 min with 5% A, *t*=6 min with 5% A, *t*=7 min with 98% A, and *t*=9 min with 98% A; (3) flow rate of 0.3 mL min^−1^; (4) column temperature 50 °C; (5) ratio of products determined by integration of spectra recorded at 210 or 254 nm; and (6) ionization mode ESI. TLC spots were detected by UV irradiation at 254 nm or with KMnO_4_ stain.

### 2.1 Synthesis of the lipid anchors

See Scheme 1.

#### 2.1.1 Preparation of ((2,3-bis((Z)-octadec-9-en-1-yloxy)propoxy)methanetriyl)tribenzene (**DIB-1**)

**Figure:**
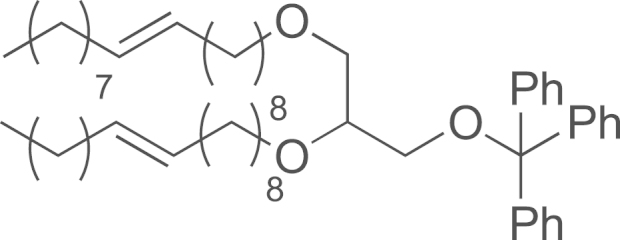

A 50% aqueous NaOH (647 mg, 16.6 mmol) solution was added to a mixture of oleyl-OMs (11) (1.15 g, 3.30 mmol), 3-(trityloxy)propane-1,2-diol (S-1) (285 mg, 0.83 mmol) and (Bu)_4_NHSO_4_ (28 mg, 0.08 mmol). The solution was stirred at 65 °C for 3 days. CH_2_Cl_2_ (50 mL) was added and the layers were separated. The aqueous layer was washed with CH_2_Cl_2_ (2×50 mL). The organic layers were combined, dried and concentrated under vacuum. The residue was purified by chromatography on silica gel using cyclohexane/EtOAc (100/0–95/5) as eluent to obtain 600 mg of ((2,3-bis((*Z*)-octadec-9-en-1-yloxy)propoxy)methanetriyl)tribenzene (**DIB-1**) as a colorless oil; ^1^H NMR (400 MHz, CDCl_3_): *δ* 7.48–7.44 (m, 6H of C_6_H_3_), 7.32–7.24 (m, 12H of C_6_H_5_), 5.41–5.35 (m, 4H), 3.58–3.53 (m, 4H), 3.43–3.39 (m, 3H), 3.19 (m, 1H) 2.02–1.91 (m, 10H), 1.38–1.20 (m, 44H), 0.90 (t, *J*=6.8 Hz, 2CH_3_); HRMS (ESI) *m*/*z* calcd. for C_58_H_90_O_3_Li^+^, 841.7050; found 841.7096.

#### 2.1.2 Preparation of ***(***Z***)***-14-***((***Z***)***-octadec-9-en-1-yloxy***)***-3***,***6***,***9***,***12***,***16-pentaoxatetratriacont-25-en-1-amine (**1**)

**Figure:**
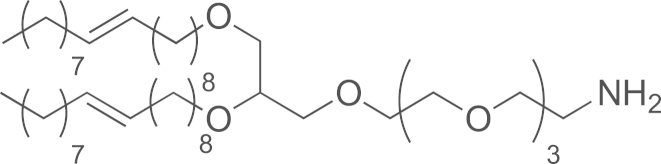

H_2_O (1 mL) was added to a mixture of (*Z*)-1-azido-14-((*Z*)-octadec-9-en-1-yloxy)-3,6,9,12,16-pentaoxatetratriacont-25-ene [2] (60 mg, 0.076 mmol) and triphenylphosphine polymer-supported (1.48 mmol/g, 155 mg, 0.23 mmol) in THF (5 mL). The mixture was shaken at 50 °C (16 h). The volatiles were evaporated and CH_2_Cl_2_ was added. The beads were filtered and placed in a filtration tube. EtOH was added to the beads and the mixture was shaken (5 min). The mixture was filtered and the beads were placed again in the filtration tube. Once again CH_2_Cl_2_ was added to the beads, the mixture was shaken (5 min) and the beads were filtered. The EtOH and CH_2_Cl_2_ washings were repeated twice. All the EtOH and CH_2_Cl_2_ layers were combined, dried and concentrated under vacuum to obtain quantitatively (*Z*)-14-((*Z*)-octadec-9-en-1-yloxy)-3,6,9,12,16-pentaoxatetratriacont-25-en-1-amine (**1**) as a colorless oil; ^1^H NMR (300 MHz, CDCl_3_): *δ* 5.35–5.30 (m, 4H), 3.64–3.40 (m, 23H), 2.05–1.90 (m, 8H), 1.60–1.52 (m, 4H), 1.40–1.19 (m, 44H), 0.86 (t, *J*=7.1 Hz, 6H).

### 2.2 Synthesis of starting materials for model reactions

#### 2.2.1 Preparation of the N-(4-chlorophenethyl)-2-(cyclooct-2-yn-1-yloxy)acetamide (**16**)

**Figure:**
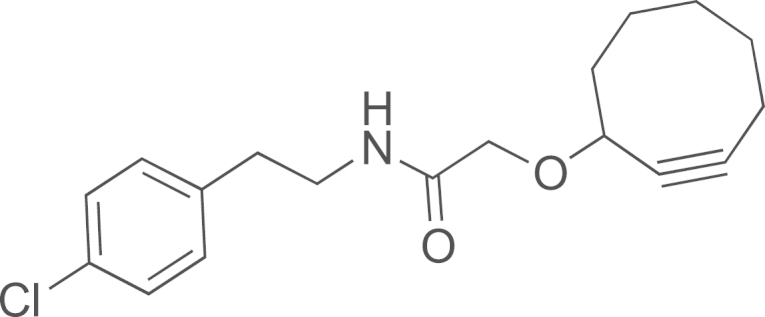

PyBOP (67 mg, 0.13 mmol) was added to a CH_2_Cl_2_ (1 mL) solution of 2-(cyclooct-2-yn-1-yloxy)acetic acid [3] (20 mg, 0.11 mmol) and DIEA (96 µL, 0.55 mmol). The solution was stirred at room temperature (30 min) and 4-chlorophenethylamine (17 µL, 0.12 mmol) was added. The solution was stirred at room temperature overnight. Then, the volatiles were evaporated and the crude was purified by flash column chromatography on silica gel with cyclohexane/EtOAc (7/3–0/10) as eluant to obtain a colorless oil (15 mg, 43%); ^1^H NMR (400 MHz, CDCl_3_): *δ* 7.21 (d, *J*=8.5 Hz, H_ar_), 7.05 (d, *J*=8.5 Hz, H_ar_), 6.50–6.41 (br s, NH), 4.12–4.07 (m, OCH), 3.94 (d, *J*=15.2 OCHH′), 3.78 (d, *J*=15.2 OCHH′), 3.45 (qd, *J*=2.0, 7.0 Hz, CH_2_N), 2.74 (t, *J*=7.0 Hz, CH_2_Ph), 2.16–2.10 (m, 2H), 2.02–1.95 (m, 1H), 1.85–1.78 (m, 3H), 1.61–1.53 (m, 2H); ^13^C NMR (100 MHz, CDCl_3_): *δ* 20.6, 25.7 (Ccyclooctyne), 28.9 (Ccyclooctyne), 26.2, 29.6, 29.7, 34.2, 35.1, 39.7, 42.2, 68.4, 73.2, 91.2, 101.8, 128.7, 130.1, 132.4, 137.2, 169.6; HRMS (ESI) *m*/*z* calcd. for C_18_H_22_ClNO_2_H^+^, 320.1411 [^35^Cl] and 322.1411 [^37^Cl]; found 320.1425 [^35^Cl] and 322.1398 [^37^Cl].

#### 2.2.2 Preparation of the N-(4-chlorophenethyl)-2-(cyclooct-2-yn-1-yloxy)-2-fluoroacetamide (**17**)

##### 2.2.2.1 Preparation of ethyl 1-fluoro-2-oxocyclooctanecarboxylate (**DIB-2**)

**Figure:**
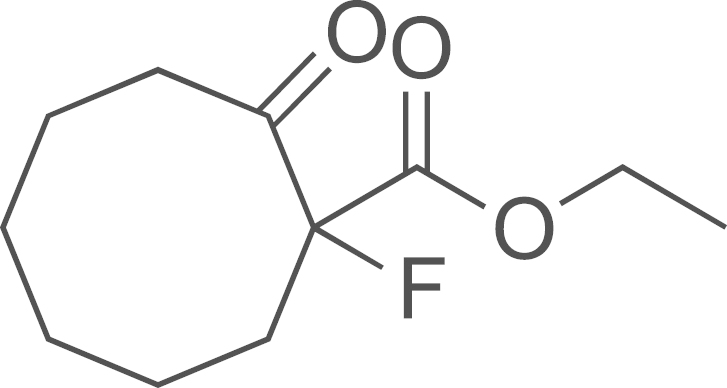

To a stirred solution of ethyl-2-oxocyclooctane-1-carboxylate (2.70 g, 13.0 mmol) in dry acetonitrile (75 ml) cooled to 0 °C was added Selectfluor (5.70 g, 16.4 mmol). The resulting mixture was then heated in a 55 °C oil bath (16 h). After cooling to room temperature the reaction was quenched with water (20 mL) and extracted with ethyl acetate (4×20 mL). The combined organic layer was dried over anhydrous Na_2_SO_4_, filtered and concentrated in vacuo to yield a clear oil (2.92 g, 99%); ^1^H NMR (400 MHz, CDCl_3_): *δ* 4.18 (q, *J*=7.2 Hz, CH_2_O), 2.67–2.46 (m, 3H), 2.20–2.16 (m, 1H), 1.94–1.91 (m, 2H), 1.83–1.55 (m, 3H), 1.46–1.33 (3H), 1.23 (t, *J*=7.2 Hz, CH_3_).

##### 2.2.2.2 Preparation of ethyl 1-fluorocyclooct-2-yne carboxylate (**DIB-3**)

**Figure:**
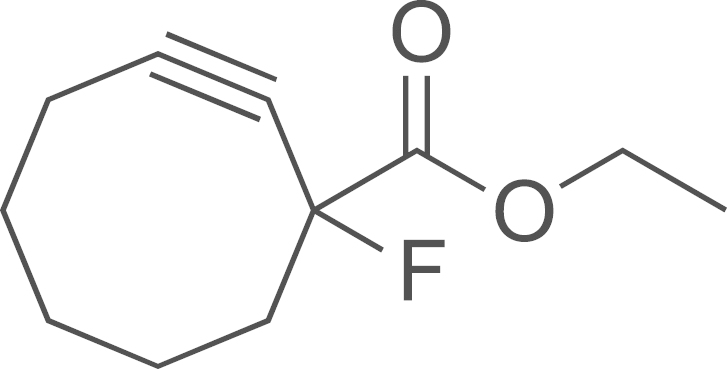

A solution of potassium hexamethyldisilazide (0.5 M in toluene, 15.6 mL, 7.8 mmol) was added dropwise to a stirred solution of **(DIB-2)** (700 mg, 3.5 mmol) in THF (40 mL) at −78 ^o^C. After the addition was complete the reaction mixture was maintained for 30 min and a THF (5 mL) solution of phenyl bis(trifluoromethane sulfonimide) (1.4 g, 3.9 mmol) was added slowly via syringe. The reaction was stirred at −78 ^o^C (1 h), and then allowed to warm up and stirred to 50 ^o^C (16 h). Methanol was added and the volatiles were evaporated under vacuum. The crude residue was purified by flash column chromatography on silica gel using 0–10% ethyl acetate in hexane to afford **(DIB-3)** as a pale yellow liquid (346 mg, 50%); ^1^H NMR (400 MHz, CDCl_3_): *δ* 4.24 (q, *J*=7.2 Hz, CH_2_O), 2.32–2.22 (m, 4H), 1.98–1.80 (m, 4H), 1.69–1.65 (m, 1H), 1.53–1.50 (m, 1H), 1.25 (t, *J*=7.2 Hz, CH_3_).

##### 2.2.2.3 Preparation of 1-fluorocyclooct-2-ynecarboxylic acid (**DIB-4**)

**Figure:**
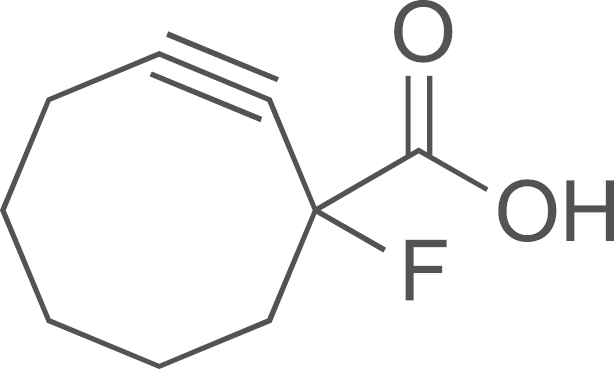

Ethyl 1-fluorocyclooct-2-yne carboxylate **(DIB-3)** (85 mg, 0.43 mmol) and LiOH (36 mg, 0.86 mmol) were combined in 4 mL of 50% aqueous MeOH. This mixture was heated at 50 ^o^C (10 min). The reaction was then allowed to cool to room temperature then stirred for additional 30 min. CH_2_Cl_2_ was added and the layers were separated. Then the aqueous layer was acidified to pH2 with a 1 M aqueous solution of HCl and was washed with ethyl acetate (3×50 mL). The ethyl acetate layers were combined, dried over anhydrous MgSO_4_, filtered, and concentrated under vacuum leading to 1-fluorocyclooct-2-ynecarboxylic acid **(DIB-4)** as colorless oil (52 mg, 70%); ^1^H NMR (400 MHz, CDCl_3_): *δ* 2.37–2.24 (m, 4H), 1.99–1.81 (m, 4H), 1.71–1.63 (m, 1H), 1.43–1.40 (m, 1H).

#### 2.2.3 Preparation of N-(4-chlorophenethyl)-1-fluorocyclooct-2-ynecarboxamide (**17**)

**Figure:**
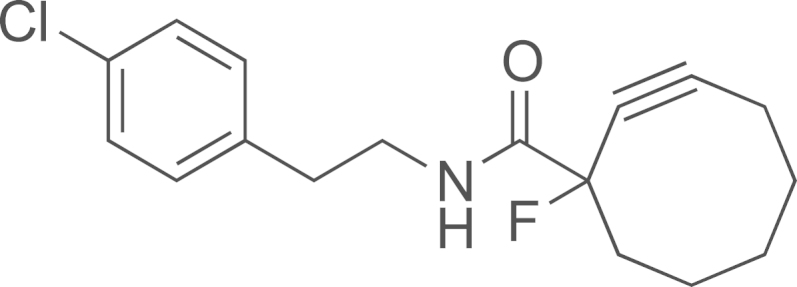

HATU (107 mg, 0.28 mmol) was added to a THF (2 mL) solution of 1-fluorocyclooct-2-ynecarboxylic acid **(DIB-4)** (40 mg, 0.24 mmol) and DIEA (81 µL, 0.47 mmol). The solution was stirred at room temperature (5 min) and the 4-chlorophenethylamine (40 µL, 0.28 mmol) was added. The solution was stirred at room temperature (16 h). The solution was concentrated under vacuum. The residue was purified by chromatographic column on silica gel using cyclohexane/EtOAc as eluent (100/0–80/20). The *N*-(4-chlorophenethyl)-1-fluorocyclooct-2-ynecarboxamide (**17**) was obtained as a white solid (40 mg, 60%);^1^H NMR (400 MHz, CDCl_3_): *δ* 7.21 (d, *J*=8.4 Hz, H_m_), 7.05 (d, *J*=8.4 Hz, H_o_), 6.30 (s, NH), 3.42–3.50 (m, CH_2_N), 2.75 (t, *J*=7.2 Hz, CH_2_Ph), 2.38–2.10 (m, 4H), 2.02–1.75 (m, 4H), 1.62–1.51 (m, 1H), 1.43–1.37 (m, 1H); ^13^C NMR (100 MHz, CDCl_3_): *δ* 20.6 (d, *J*=2.9 Hz), 25.7, 28.9, 33.9, 34.8, 40.5 (d, *J*=7.2 Hz), 46.3 (d, *J*=24.8 Hz, Ccyclooctyne), 87.1 (d, *J*=32.1 Hz), 94.5 (d, *J*=184.5 Hz), 109.5 (d, *J*=10.9 Hz), 128.8, 130.1, 132.5, 136.9, 168.3 (d, *J*=24.8 Hz, C(O)); MS (ESI) *m*/*z* calcd. for C_17_H_19_ClFNOH^+^, 307.1 [^35^Cl] and 309.1 [^37^Cl]; found 308.0 [^35^Cl] and 310.0 [^37^Cl].

#### 2.2.4 Preparation of the methyl 4-((4-chlorophenethyl)carbamoyl)-2-(diphenylphosphino)benzoate (**18**)

**Figure:**
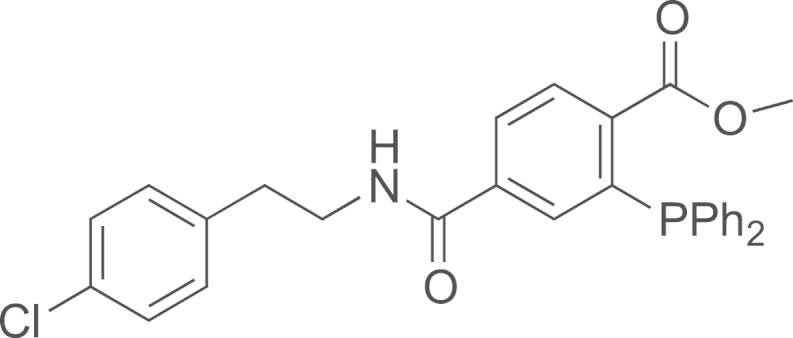

4-chlorophenethylamine (8 µL, 0.056 mmol) was added to a DMF (1 mL) solution of 2-(diphenylphosphino)terephthalic acid 1-methyl 4-pentafluorophenyl diester (25 mg, 0.047 mmol) and Et_3_N (33 µL, 0.235 mmol). The solution was stirred at room temperature overnight. The solution was concentrated under vacuum and the residue was purified by chromatographic column on silica gel using cyclohexane/EtOAc as eluent (100/0–80/20). The methyl 4-((4-chlorophenethyl)carbamoyl)-2-(diphenylphosphino)benzoate (**18**) was obtained as yellow oil (19 mg, 82%); ^1^H NMR (500 MHz, CDCl_3_): *δ* 8.00 (dd, *J*=3.5, 8.0 Hz, 1 H_ar_), 7.65 (dd, *J*=1.5, 8.0 Hz, 1 H_ar_), 7.28–7.24 (m, 6 H_ar_), 7.21–7.10 (m, 6 H_ar_), 7.09–7.10 (m, 1 H_ar_), 6.97 (d, *J*=8.5 Hz, 2 H_ar_), 5.70–5.60 (br m, NH), 3.67 (s, OCH_3_), 3.50 (q, *J*=7.0 Hz, CH_2_N), 2.70 (t, *J*=7.0 Hz, CH_**2**_Ph); ^13^C NMR (133 MHz, CDCl_3_): *δ* 34.8, 41.0, 52.3, 127.0, 128.6, 128.7, 129.0, 130.0 131.0, 131.1, 132.1, 132.5, 133.8, 133.9, 137.0, 137.1, 166.5, 166.6; ^31^P NMR (500 MHz, CDCl_3_): *δ* 3.98; HRMS (ESI) *m*/*z* calcd. for C_29_H_25_ClNO_3_P(O)H^+^, 518.1288 [^35^Cl] and 520.1288 [^37^Cl]; found 518.12969 [^35^Cl] and 520.1279 [^37^Cl].

#### 2.2.5 Synthesis of biotin-OSuc (**14**) [4]

**Figure:**
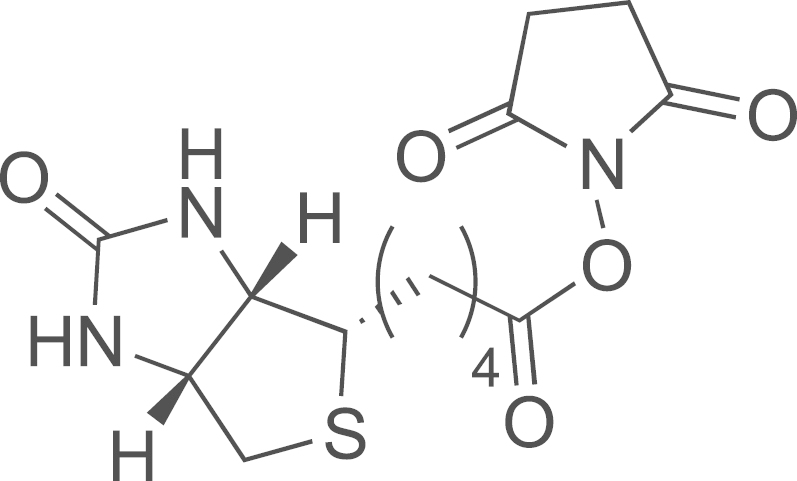

A mixture of biotin (2.5 g, 12.3 mmol), N-hydroxysuccinimide (2.0 g, 16.25 mmol), and EDCI (2.5 g, 13.30 mmol) was dissolved in DMF (40 mL) and stirred for 24 h at room temperature. The solution was poured onto crushed ice and the solid obtained was filtered, washed with water and dried under vacuum to give Biotin-OSuc (2.6 g, quant.yield); ^1^H NMR (300 MHz, DMSO-d_6_): *δ* 6.43 (s, 1H), 6.37 (s, 1H), 4.33–4.29 (m, 1H), 4.17–4.12 (m, 1H), 3.09–3.12 (m, 1H), 2.89–2.78 (m, 6H), 2.67 (t, *J*=7.4 Hz, 2H), 2.58 (d, *J*=12.4Hz, 1H), 1.66–1.60 (m, 3H), 1.53–1.41 (m, 3H).

##### 2.2.5.1 Preparation of 2-bromo-N-(4-chlorophenethyl)acetamide (**DIB-5**)

**Figure:**
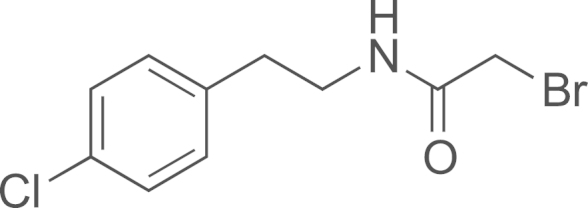

Bromoacetyl bromide (0.672 mL, 7.71 mmol) was added dropwise at 0 °C to a solution of 4-chlorophenethylamine (0.9 mL, 6.43 mmol) and triethylamine (1.8 mL, 12.86 mmol) in CH_2_Cl_2_ (24 mL). The solution was stirred for 2 h, then water (25 mL) was added and the product was extracted in the organic phase, which was washed with HCl 1 M. After evaporation, the crude was purified by flash chromatography on silica gel (cyclohexane/AcOEt gradient 10:0–5:5) to yield 2-bromo-N-(4-chlorophenethyl)acetamide (1.06 g, 60% yield) as a yellow solid; ^1^H NMR (400 MHz, CDCl_3_): *δ* 7.31 (d, *J*=8.2 Hz, 2H), 7.15 (d, *J*=8.2 Hz, 2H), 6.50 (bs, 1H), 3.86 (s, 2H), 3.53 (app q, *J*=6.9 Hz, 2H), 2.83 (t, *J*=6.9 Hz, 2H).

#### 2.2.6 Preparation of N-(4-chlorophenethyl)-2-(3,4-dibromo-2,5-dioxo-2,5-dihydro-1H-pyrrol-1-yl)acetamide (**19**)

**Figure:**
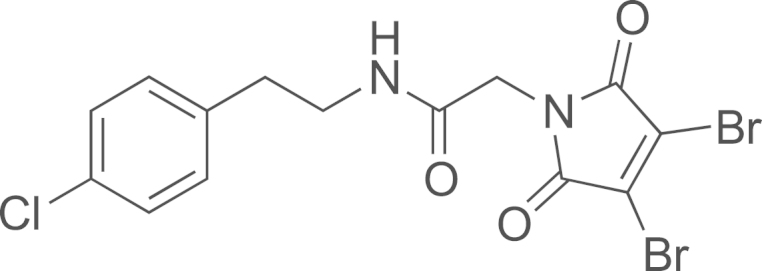

Dibromomaleimide (92 mg, 0.36 mmol) and K_2_CO_3_ (5 mg, 0,036 mmol) were added to a solution of 2-bromo-N-(4-chlorophenethyl)acetamide (**DIB-5**)(50 mg, 0.18 mmol) in acetone (5 mL). The mixture was stirred at room temperature for three days, then the solvent was distilled and the crude was purified on silica gel (cyclohexane/AcOEt 7:3) to afford N-(4-chlorophenethyl)-2-(3,4-dibromo-2,5-dioxo-2,5-dihydro-1H-pyrrol-1-yl)acetamide (1.06 g, 70% yield) as a yellow solid; ^1^H NMR (400 MHz, CDCl_3_): δ 7.31 (d, *J*=8.4 Hz, 2H), 7.14 (d, *J*=8.4 Hz, 2H), 5.59 (bs, 1H), 3.86 (s, 2H), 3.53 (app q, *J*=7.1 Hz, 2H), 2.83 (t, *J*=7.1 Hz, 2H).

### 2.3 Ligation reactions

See Scheme 2.

#### 2.3.1 Preparation of N-(4-chlorophenethyl)-2-((1-(11-hydroxyundecyl)-4,5,6,7,8,9-hexahydro-1H-cycloocta[d][1,2,3]triazol-4-yl)oxy)acetamide and N-(4-chlorophenethyl)-2-((1-(11-hydroxyundecyl)-4,5,6,7,8,9-hexahydro-1H-cycloocta[d][1,2,3]riazol-9-yl)oxy)acetamide (20)

**Figure:**
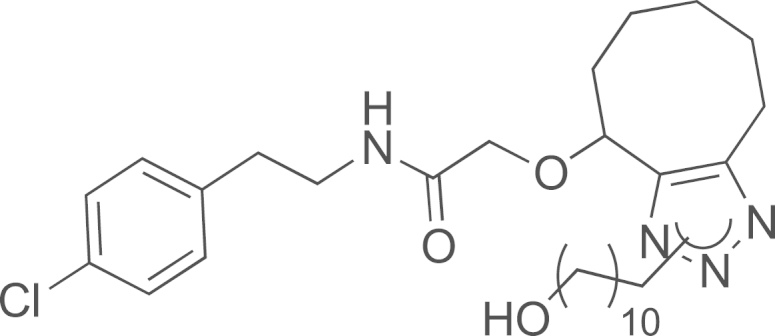

*N*-(4-chlorophenethyl)-2-(cyclooct-2-yn-1-yloxy)acetamide **16** (10 mg, 0.030 mmol) was added to a methanol (1 mL) solution of 11-azidoundecan-1-ol (6.7 mg, 0.031 mmol). The solution was stirred at room temperature (3 h). Then, the volatiles were evaporated and the crude was purified on silica gel using cyclohexane/EtOAc (50/50–20/80) to obtain the expected compound as a colorless oil corresponding to a mixture of regioisomers **20** (11.0 mg, 68%); ^1^H NMR (400 MHz, CDCl_3_): *δ* 7.69–7.62 (br m, 0.5H, 0.5 NH), 7.30–7.23 (m, 2H, H_ar_), 7.10–7.20 (m, 2H, H_ar_), 6.41–6.76 (br m, 0.5H, 0.5 NH), 4.71–4.65 (m, 1H), 4.12–4.25 (m, 2H), 4.03 (d, 1H, *J*=15.7 Hz, CHH′O), 4.03 (d, 1H, *J*=15.7 Hz, CHH′O), 3.67–3.51 (m, 4H), 2.92–2.82 (m, 3H), 2.75–2.68 (m, 4H, 2 CH_2_), 1.87–1.64 (m, 2H, CH_2_), 1.61–1.56 (m, 6H), 1.33–1.25 (m, 18H); ^13^C NMR (100 MHz, CDCl_3_): *δ* 20.8, 22.0, 22.3, 24.7, 25.7, 26.7, 28.9, 29.0, 29.3, 29.3, 29.4, 29.7, 30.3, 30.4, 32.6, 30.4, 32.6, 32.8, 34.8, 34.9, 39.7, 39.8, 47.8, 63.0, 63.0, 67.5, 68.5, 75.4, 128.6, 128.8, 130.0, 130.2, 137.5, 170.1; MS (ESI) m/z calcd for C_29_H_45_ClN_4_O_3_H^+^ 533.3258 [^35^Cl], 535.3258 [^37^Cl]; found 533.3247 [^35^Cl], 535.3226 [^37^Cl].

#### 2.3.2 Preparation of N-(4-chlorophenethyl)-4-fluoro-1-(11-hydroxyundecyl)-4,5,6,7,8,9-hexahydro-1H-cycloocta[d][1,2,3]triazole-4-carboxamide and N-(4-chlorophenethyl)-9-fluoro-1-(11-hydroxyundecyl)-4,5,6,7,8,9-hexahydro-1H-cycloocta[d][1,2,3]triazole-9-carboxamide (**21**)

**Figure:**
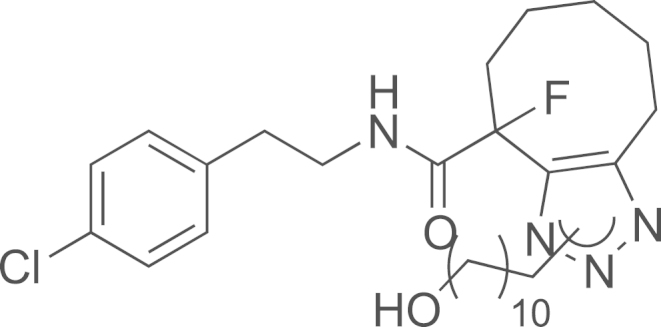

*N*-(4-chlorophenethyl)-1-fluorocyclooct-2-yne carboxamide **17** (8.6 mg, 0.028 mmol) was added to a methanol (1 mL) solution of 11-azidoundecan-1-ol (6.0 mg, 0.028 mmol). The solution was stirred at room temperature (5 h). Then, the volatiles were evaporated and the crude was purified on silica gel using cyclohexane/EtOAc (50/50–20/80) to obtain the expected compound as a colorless oil corresponding to a mixture of regioisomers **21** (9.0 mg, 62%); ^1^H NMR (400 MHz, CDCl_3_): *δ* 7.36–7.32 (m, 2H, 2 H_ar_), 7.20–7.13 (m, 2H, 2 H_ar_), 6.98–7.00 (br s, 0.5H, 0.5 NH), 6.54 (s, 0.5H, 0.5 NH), 4. 26–4.02 (m, 2H, CH_2_), 3.66–3.48 (m, 4H, 2 CH_2_), 2.95–2.82 (m, 4H, 2 CH_2_), 1.82–1.43 (m, 8H), 1.30–1.26 (m, 18H); ^13^C NMR (100 MHz, CDCl_3_) *δ* (ppm): 21.4, 22.1, 22.1, 22.5, 22.6, 23.3, 24.0, 25.1, 25.7, 26.1, 26.2, 26.5, 26.7, 28.9, 29.0, 29.3, 29.3, 29.3, 29.4, 29.8, 30.4, 32.8, 33.1, 33.4, 34.8, 34.8, 35.0, 40.4, 40.5, 48.0, 49.8, 49.9, 63.0, 128.7, 129.0, 130.0, 130.3, 132.8, 136.4, 137.2, 142.3, 142.5, 145.8, 145.9; MS (ESI) *m*/*z* calcd. for C_28_H_42_ClFN_4_O_2_H^+^ 521.30; found 521.20.

#### 2.3.3 Preparation of N-(4-chlorophenethyl)-2-(diphenylphosphoryl)-N′-(11-hydroxyundecyl)terephthalamide (**22**)

**Figure:**
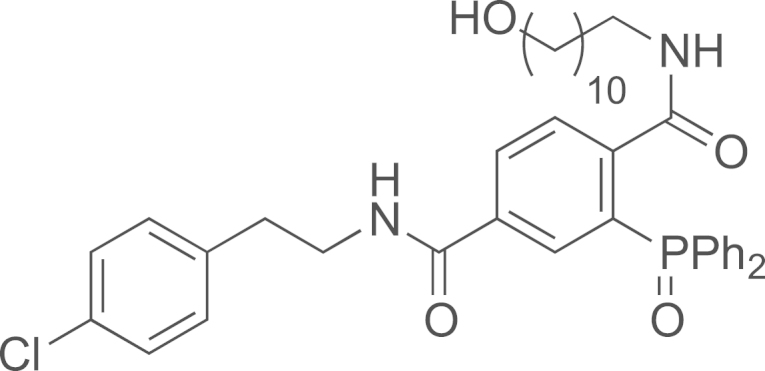

1-Azido-11-hydroxyundecane (4.2 mg, 0.02 mmol) was added to a THF/H_2_O (600 µL/200 µL) solution of methyl 4-((4-chlorophenethyl)carbamoyl)-2-(diphenylphosphino)benzoate **18** (10 mg, 0.02 mmol). The solution was stirred at room temperature (16 h). The volatiles were evaporated and the *N*-(4-chlorophenethyl)-2-(diphenylphosphoryl)-*N*′-(11-hydroxyundecyl)terephthalamide (**22**) was obtained as a colorless oil (13 mg, quant.); ^1^H NMR (400 MHz, CDCl_3_): δ 7.80–7.86 (m, 2H, 2 H_ar_), 7.42–7.61 (m, 12H, 12 H_ar_), 7.19 (d, 2H, *J*=7.8 Hz, 2 H_ar_), 7.02 (d, 2H, *J*=7.8 Hz, 2 H_ar_), 3.50–3.58 (m, 5H), 3.18 (t, *J*=7.0 Hz, 1H), 2.76 (t, 4H, *J*=7.0 Hz, 2 CH_2_), 1.46–1.50 (m, 5H), 1.21–1.15 (m, 12H); ^13^C NMR (100 MHz, CDCl_3_) *δ* (ppm): 24.7, 25.7, 25.9, 27.5, 27.8, 28.0, 28.30, 28.32, 28.34, 28.38, 28.4, 28.5, 28.52, 31.8, 33.8, 39.3, 40.1, 62.0, 62.1, 127.7, 127.8, 127.90, 128.94, 129.0, 129.2, 130.0, 130.6, 130.7, 130.74, 130.8, 130.9, 131.2, 131.3, 131.5, 131.6, 161.7, 136.0, 164.6, 165.5; MS (ESI) *m*/*z* calcd for C_39_H_46_ClN_2_O_4_PH^+^ 673.2961 [^35^Cl], 675.2961 [^37^Cl]; found 673.2976 [^35^Cl], 675.2963 [^37^Cl].

#### 2.3.4 Preparation of *N*-(4-chlorophenethyl)-2-(5,7-dioxotetrahydro-2*H*-[1,4]dithiino[2,3–c]pyrrol-6(3*H*)-yl)acetamide (**23**)

**Figure:**
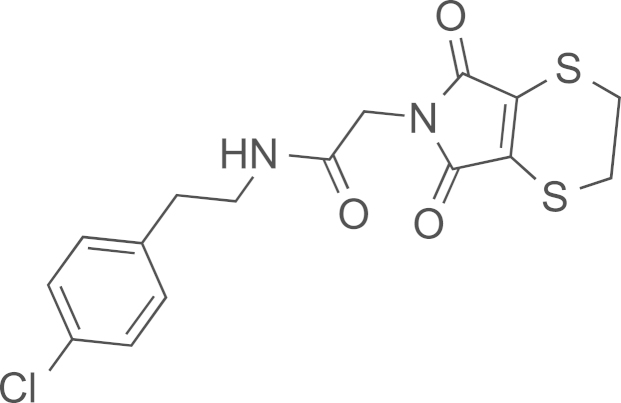

TCEP (10 mg, 0.04 mmol) and 1,2-ethanedithiol (24 mg, 0.04 mmol) were added to a CH_2_Cl_2_ (1 mL) solution of *N*-(4-chlorophenethyl)-2-(3,4-dibromo-2,5-dioxo-2,5-dihydro-1 H-pyrrol-1-yl)acetamide **19** (9 mg, 0.02 mmol). The mixture was stirred at room temperature (16 h). H_2_O (2 mL) was added and the layers were separated. The organic layer was evaporated to obtained **23** (50% yield after silica gel column with cyclohexane/EtOAc 50/50–20/80); MS (ESI) *m*/*z* calcd. for C_16_H_15_ClN_2_O_3_S_2_H^+^ 383.0213 [^35^Cl]; found 383.0283 [^35^Cl].

## Figures and Tables

**Scheme 1:**
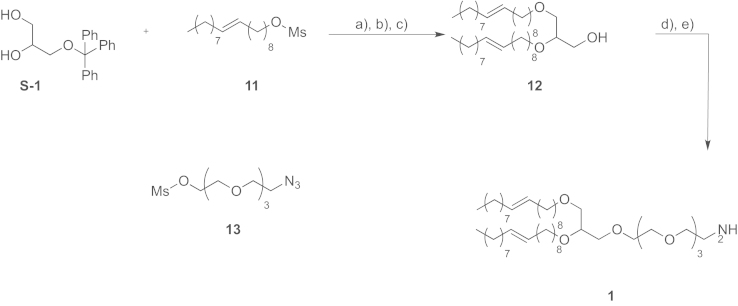
Synthesis of DOG-PEG_3_-NH_2_: (a) **11**, TBAS 0.1%, NaOH, H_2_O, (b) APTS, THF/MeOH (1/1), (c) **13**, NaH (60%), THF, HMPA, and (d) polymer-bound PPh_3_, H_2_O, THF.

**Scheme 2:**
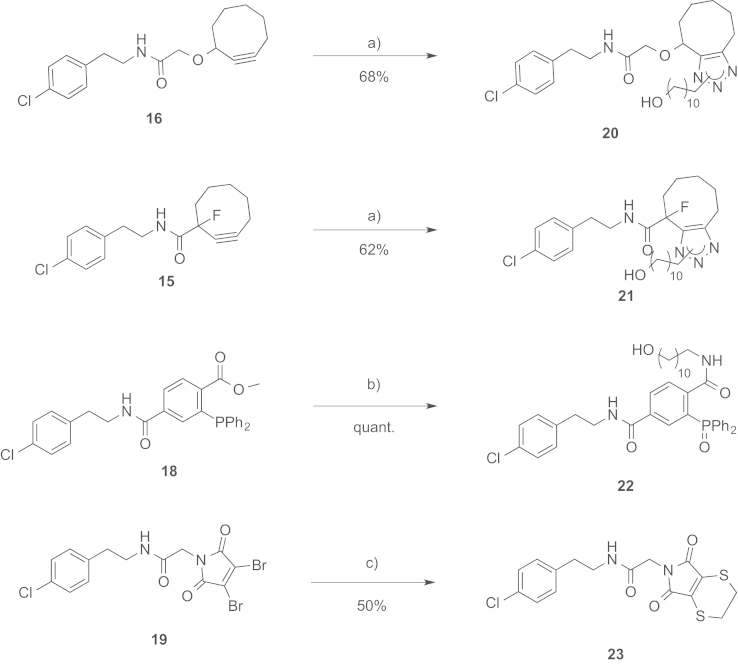
In solution evaluation of the chemical baits: (a) 11-azidoundecan-1-ol, MeOH, rt, (b) 11-azidoundecan-1-ol, THF/H_2_O (3/1), and (c) 1,2-ethanedithiol, TCEP, CH_2_Cl_2_.
