# Supplementary material for: Smart tools and orthogonal click-like reactions onto small unilamellar vesicles: Additional molecular data
Source: Data Brief. 2015 Aug 28;5:145–54. doi: 10.1016/j.dib.2015.08.014 (PMC4588400; doi:10.1016/j.dib.2015.08.014)
Supplement: Supplementary file 1 — Supplementary data [file mmc1.doc]

**Smart Tools and Orthogonal Click-like Reactions onto Small Unilamellar Vesicles. Additional Molecular Data.**

**Maria Vittoria Spanedda, Christophe Salomé, Benoit Hilbold, Etienne Berner, Béatrice Heurtault, Sylvie Fournel, Benoit Frisch* and Line Bourel-Bonnet***

*Laboratoire de Conception et Application de Molécules Bioactives, Equipe de BioVectorologie, UMR 7199 - CNRS / Université de Strasbourg, Faculté de Pharmacie, 74 route du Rhin, BP 60024, 67401 Illkirch Cedex, France.*

***Address for correspondence**: Benoît Frisch, [frisch@unistra.fr](mailto:Frisch@unistra.fr), tel : +33-(0)368-854-168 and Line Bourel, tel: +33-(0)368-854-143, fax: +33-(0)368-854-306; e-mail: [line.bourel@unistra.fr](mailto:line.bourel)

**Keywords:** liposome, click chemistry, copper-free, biotin, double ligation, Staudinger-Bertozzi, dibromomaleimide

**Declaration of interest**

The authors report no competing financial interest.
