# Supplementary material for: Smart tools and orthogonal click-like reactions onto small unilamellar vesicles: Additional molecular data
Source: Data Brief. 2015 Aug 28;5:145–54. doi: 10.1016/j.dib.2015.08.014 (PMC4588400; doi:10.1016/j.dib.2015.08.014)
Supplement: Supplementary file 2 — Supplementary data [file mmc2.pdf]

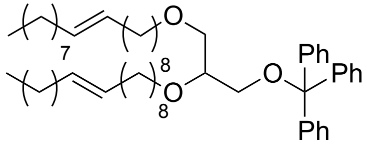

DIB-1

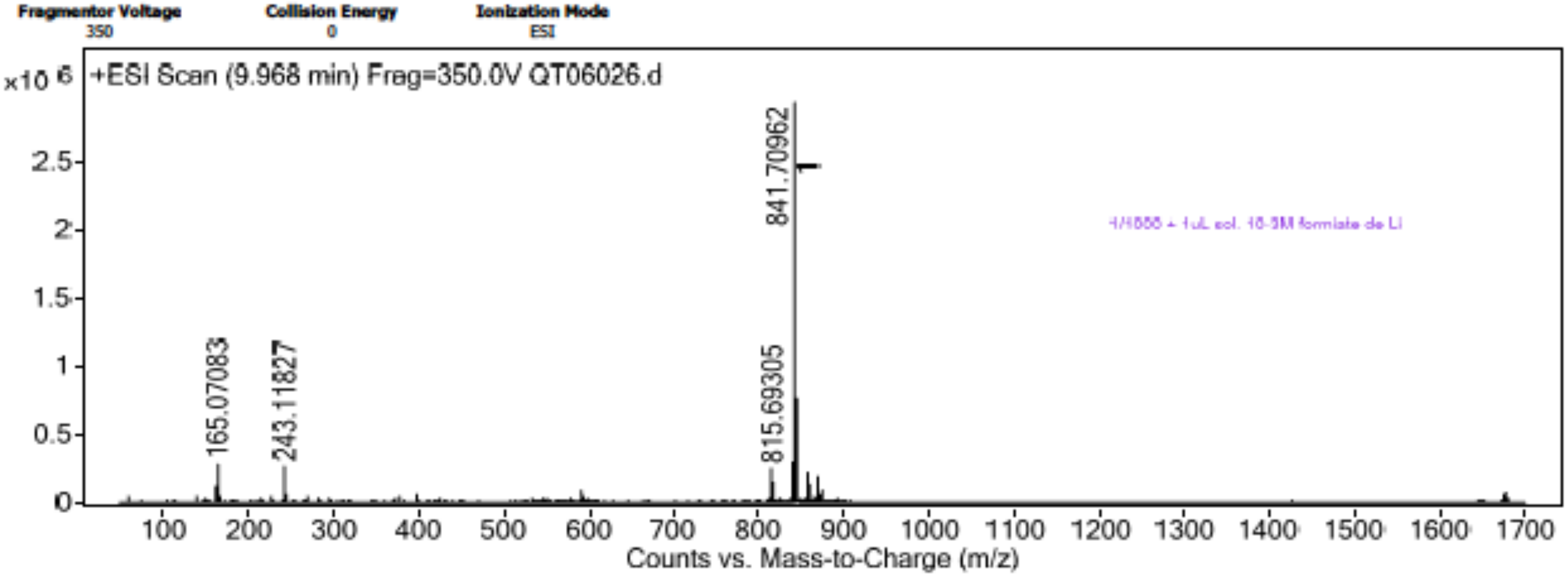

Peak List

m/z	z	Abund	Formula	Ion
165.07083		287348.4		
243.11827		270618.1		
815.69305		249725.9		
840.70835	1	295676.1		
841.70962	1	2968683.7	C58 H90 [7Li] O3	M+
842.71286	1	1935566.3	C58 H90 [7Li] O3	M+
843.71809	1	784919.6	C58 H90 [7Li] O3	M+
844.72229	1	225746.9	C58 H90 [7Li] O3	M+
857.68874		217862.6		
869.74021		195680.3		

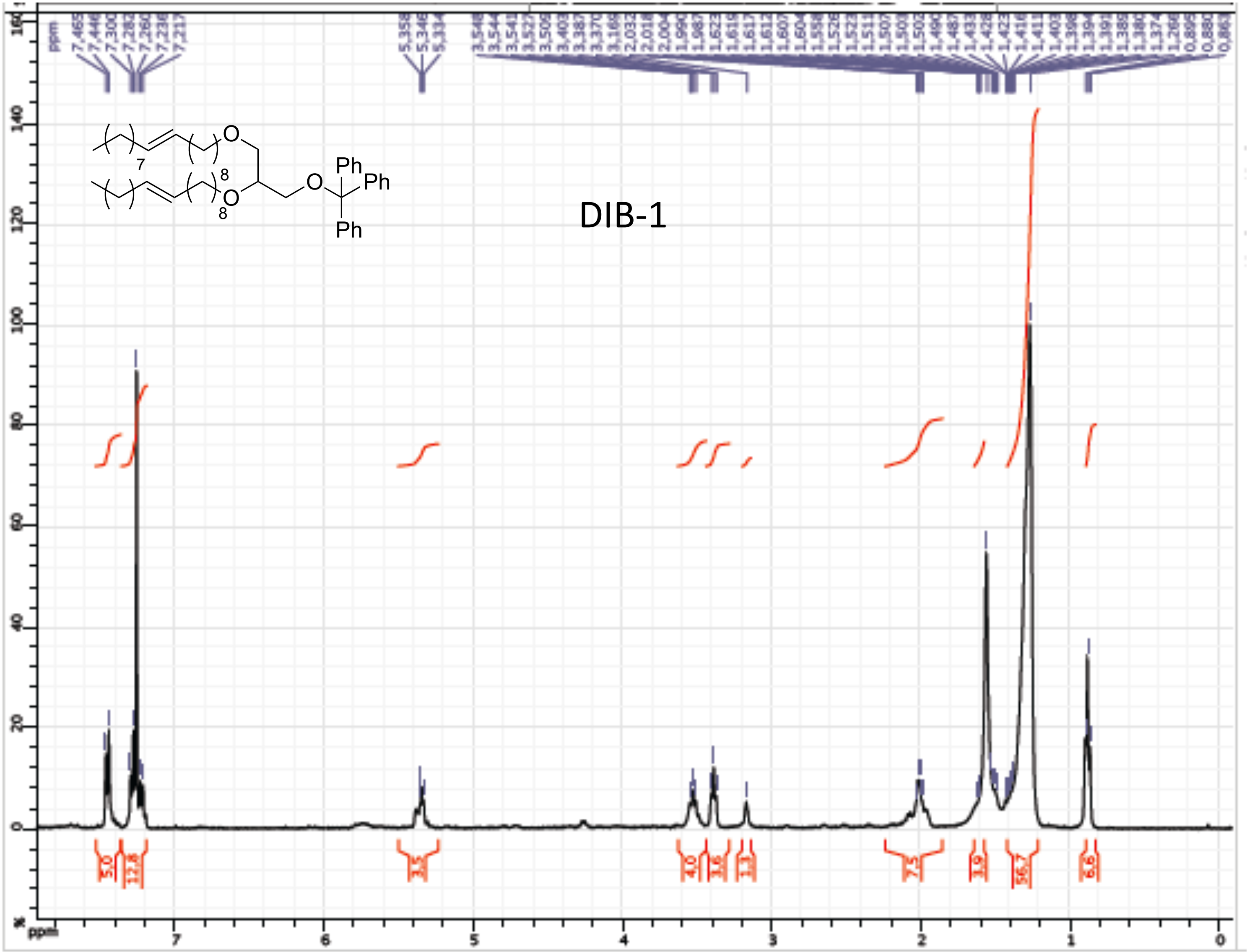
DIB-1
Ph
Ph
Ph
ppm

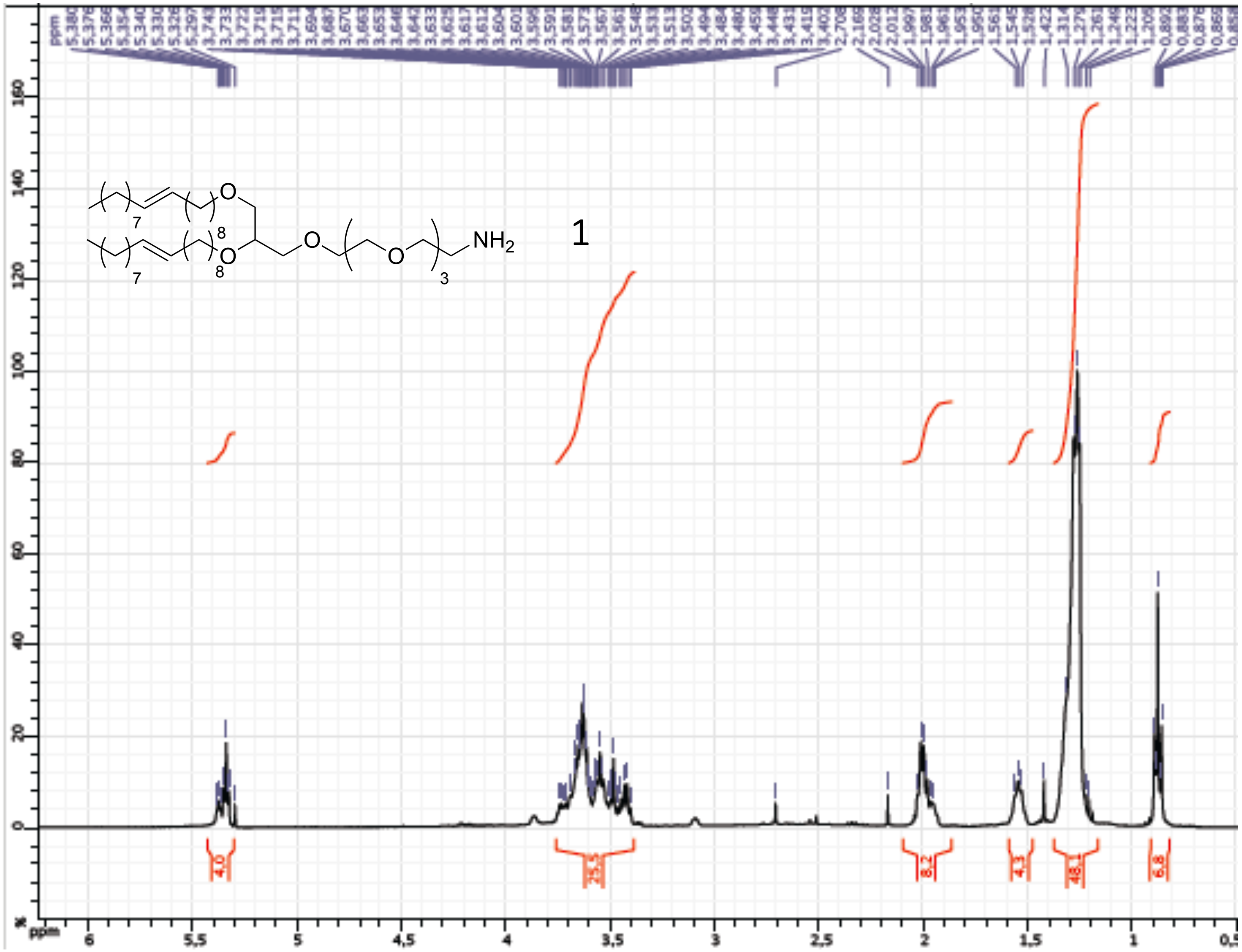

ppm
1
NH2
4,0
25,5
8,2
4,3
48,1
6,8

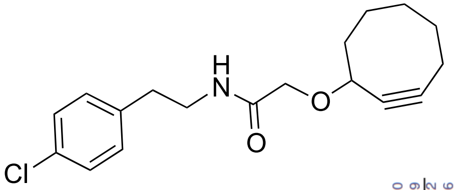

16

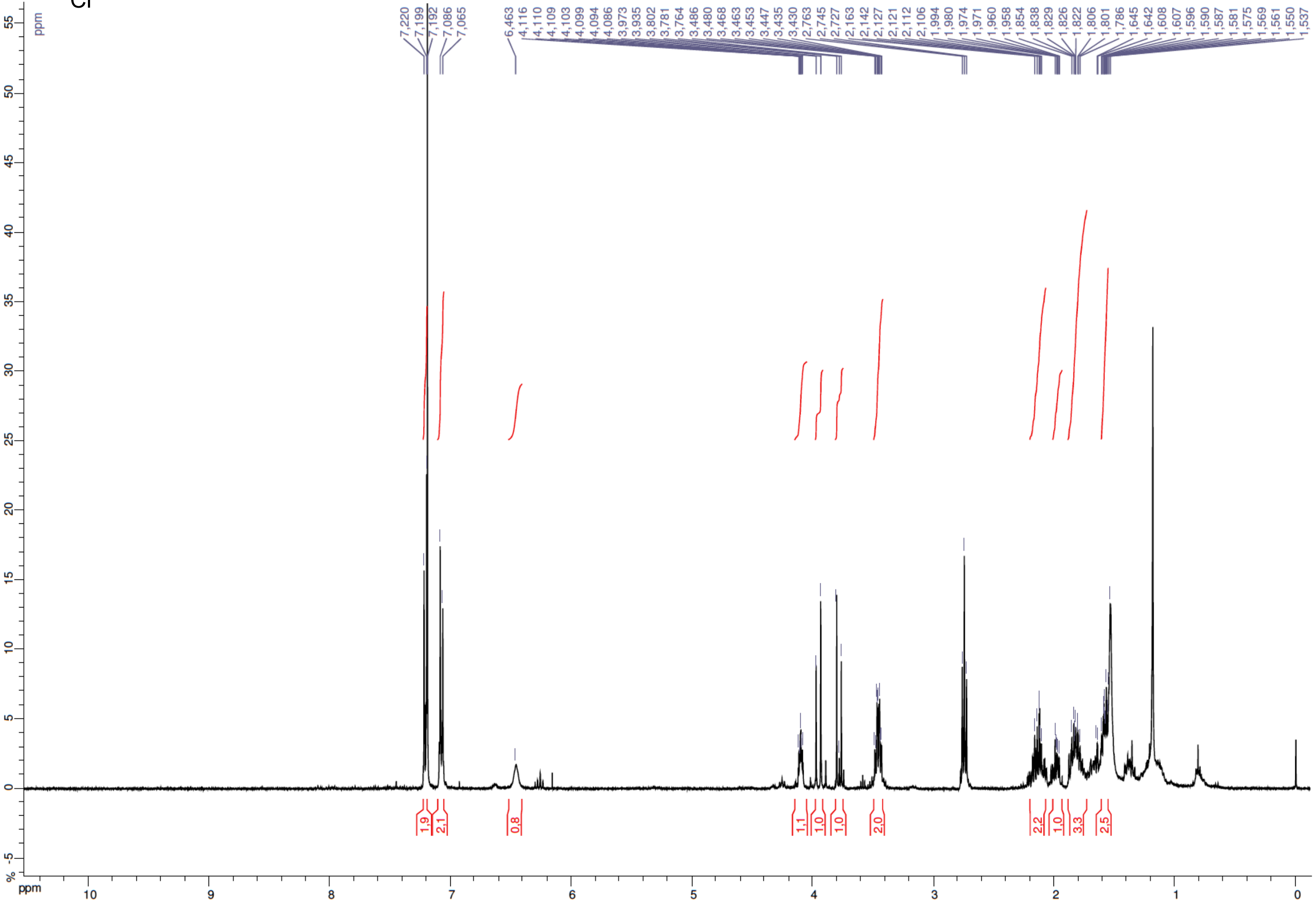

7,220
7,199
7,192
7,086
7,065
6,463
4,116
4,110
4,109
4,103
4,099
4,094
4,086
3,973
3,935
3,802
3,781
3,764
3,486
3,480
3,468
3,463
3,453
3,447
3,435
3,430
2,763
2,745
2,727
2,163
2,142
2,127
2,121
2,112
2,106
1,994
1,980
1,974
1,971
1,960
1,958
1,854
1,838
1,829
1,826
1,822
1,806
1,801
1,786
1,645
1,642
1,608
1,607
1,596
1,590
1,587
1,581
1,575
1,569
1,561
1,550
1,537
ppm
1,9
2,1
0,8
1,1
1,0
1,0
2,0
2,2
1,0
3,3
2,5
ppm
10
9
8
7
6
5
4
3
2
1
0
%

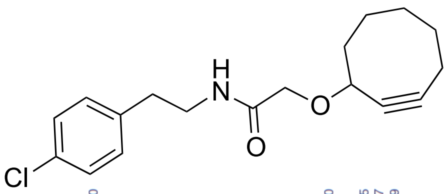

16

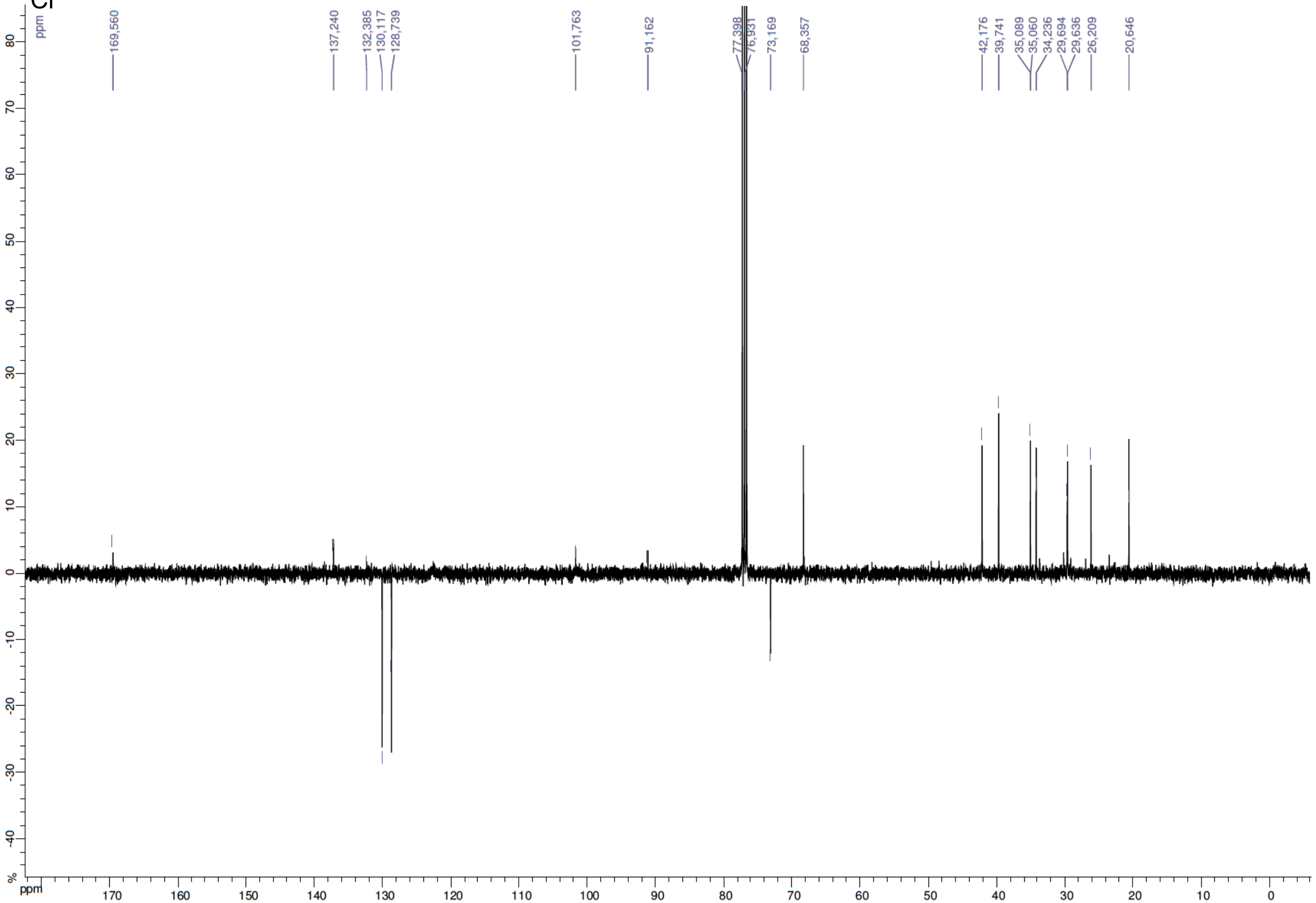

ppm
169,560
137,240
132,385
130,117
128,739
101,763
91,162
77,398
76,931
73,169
68,357
42,176
39,741
35,089
35,060
34,236
29,694
29,636
26,209
20,646
%
ppm

Spectrum Source	Fragmentor Voltage	Collision Energy	Ionization Mode
Peak (4) in "+ BPC(50.00000-1000.00000 [-41]) Scan"	120	0	ESI

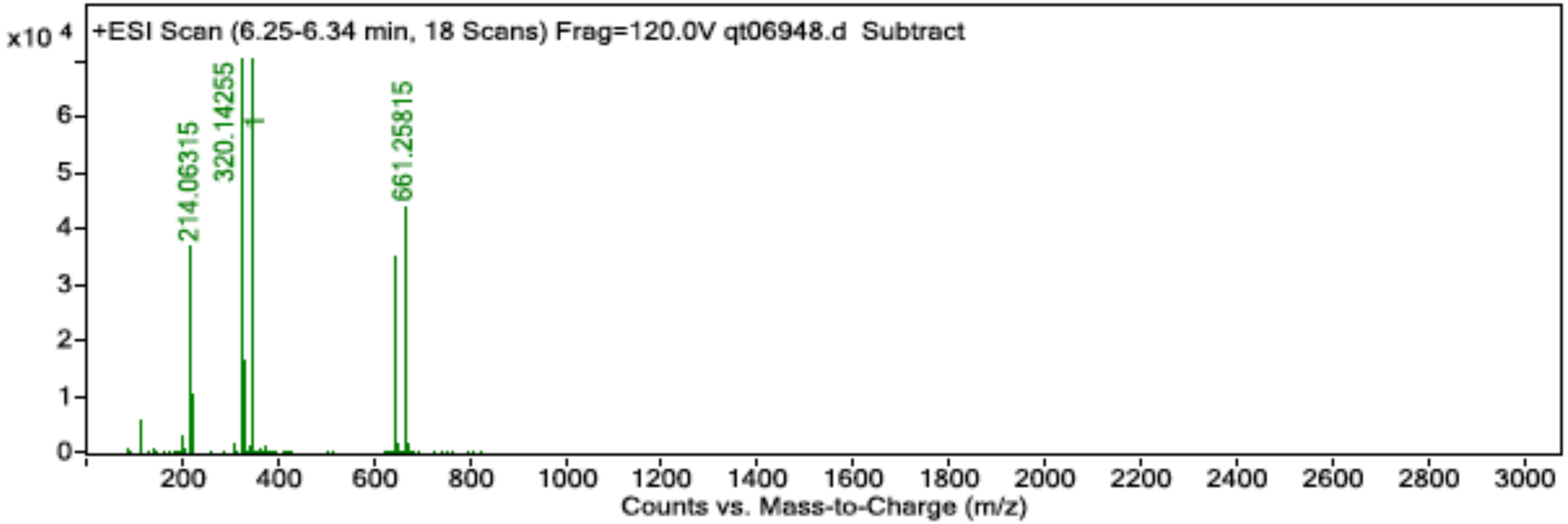

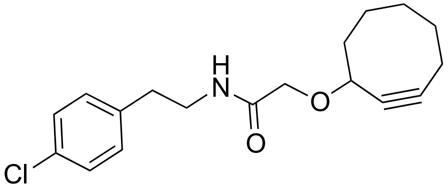

16

Peak List

m/z	z	Abund	Formula	Ion
214.06315		36869.7		
320.14255	1	323585.1		
321.14526	1	57313.8		
322.1398	1	99718.2		
342.12407		71324.6	C18 H22 Cl N Na O2	(M+Na)+
344.12146		20876.1		
639.2762		35384.7		
641.27422		24402.6		
661.25815		44096.4		
663.25635		30373.9		

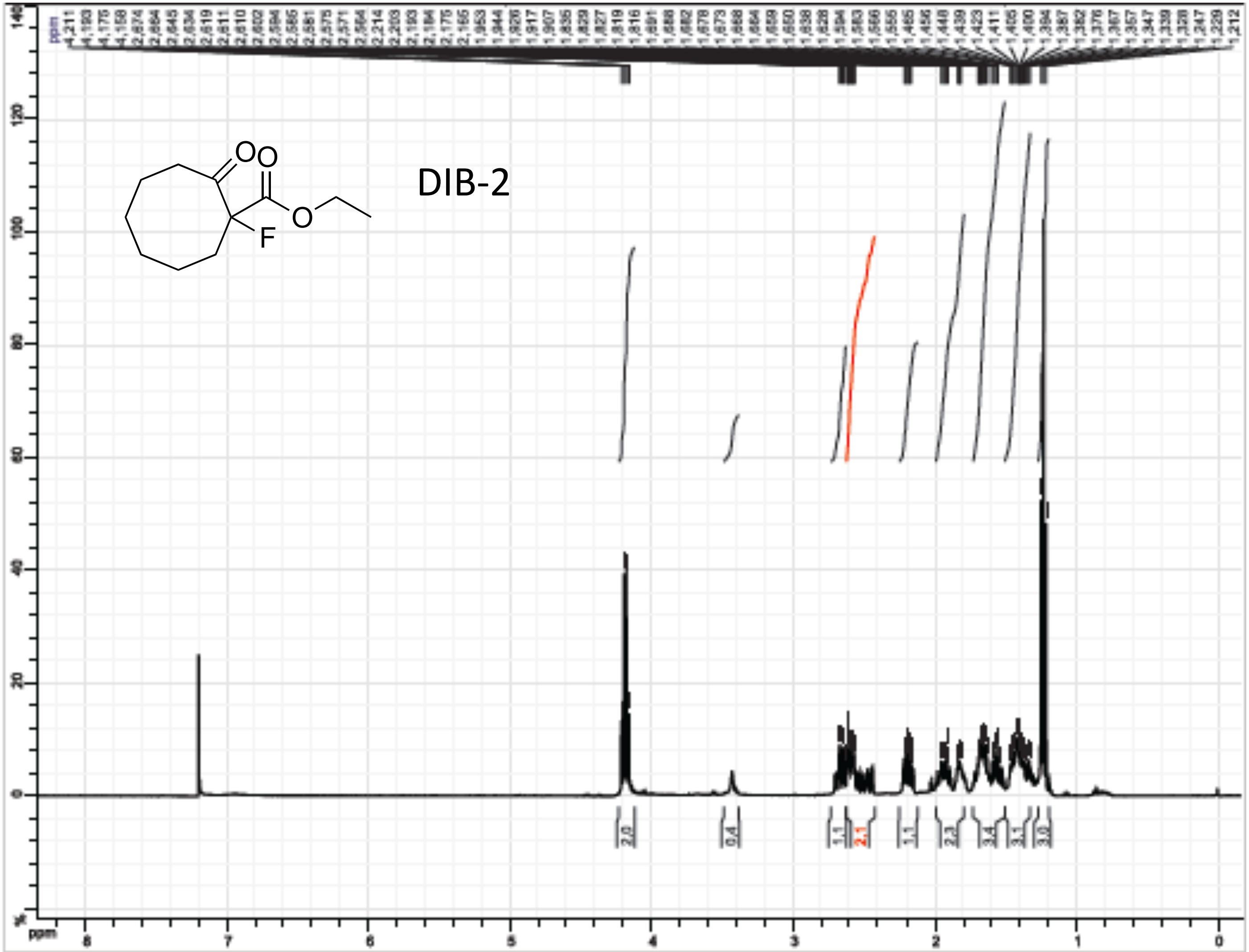
DIB-2
O
O
O
F
ppm
2.0
0.4
1.1
2.1
1.1
2.3
3.4
3.1
3.0

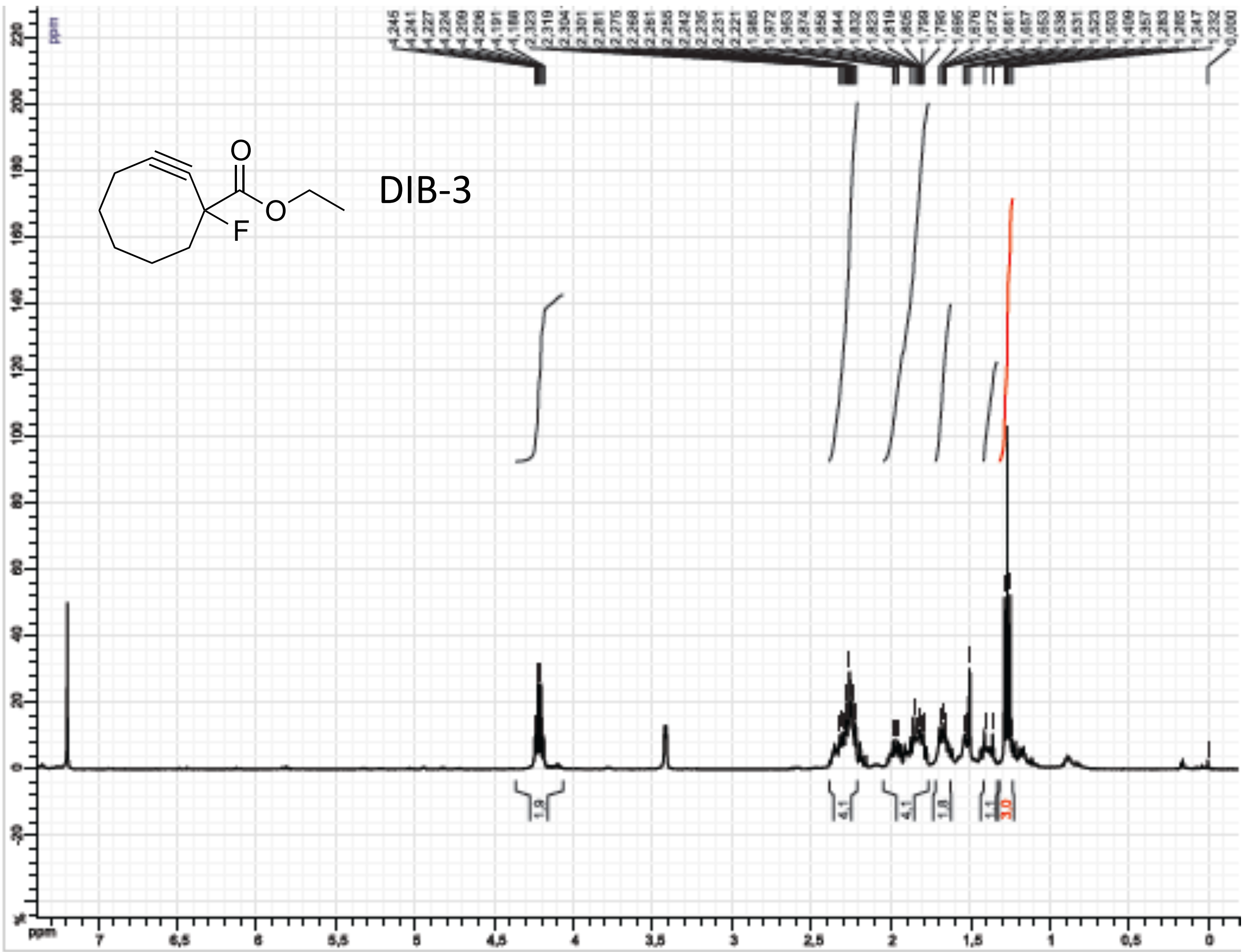
DIB-3
ppm

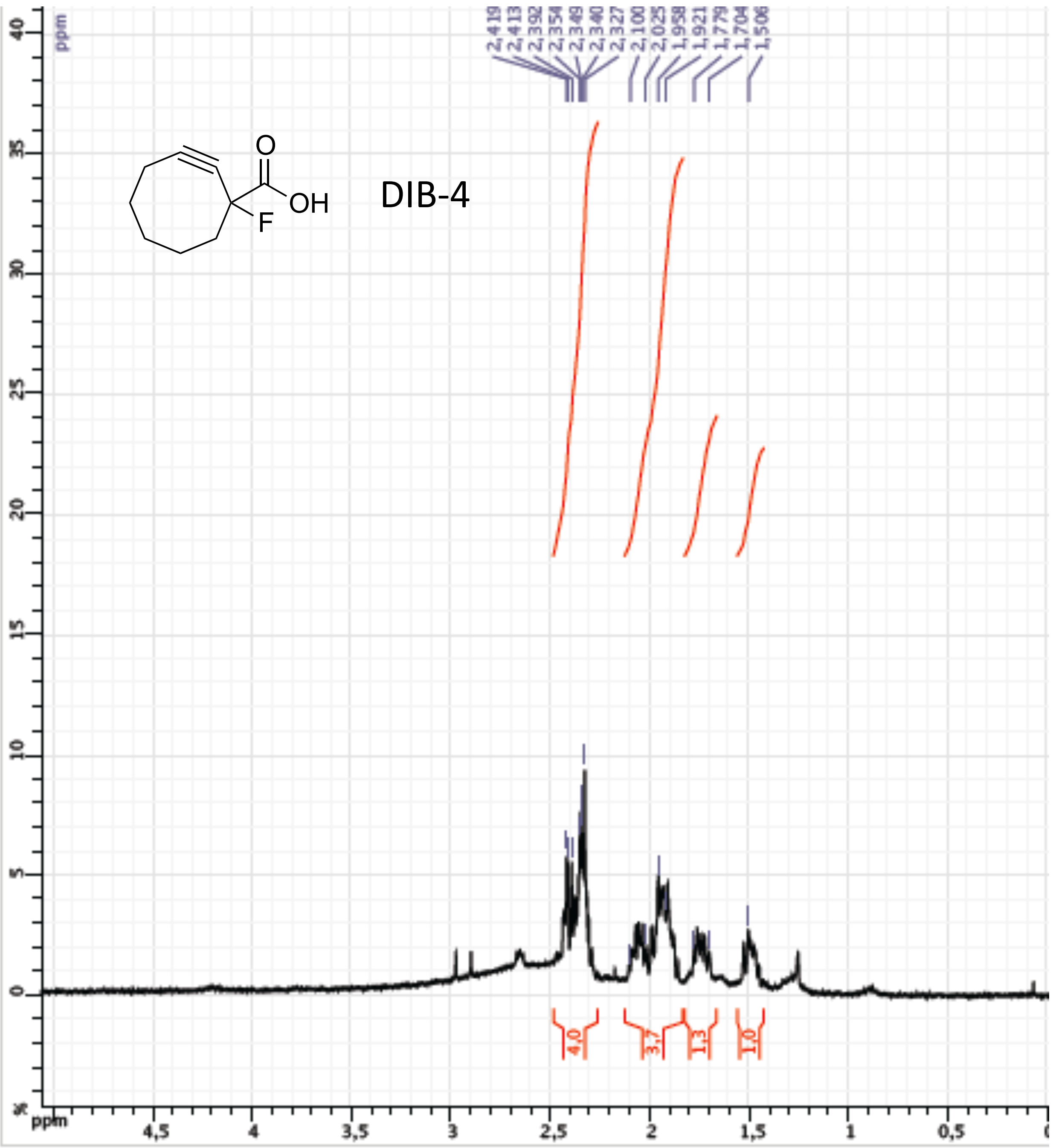

DIB-4
O
OH
F
ppm
2,419
2,413
2,392
2,354
2,349
2,340
2,327
2,100
2,025
1,958
1,921
1,779
1,704
1,506
4,0
3,7
1,3
1,0
%
40
35
30
25
20
15
10
5
0
ppm
4,5
4
3,5
3
2,5
2
1,5
1
0,5

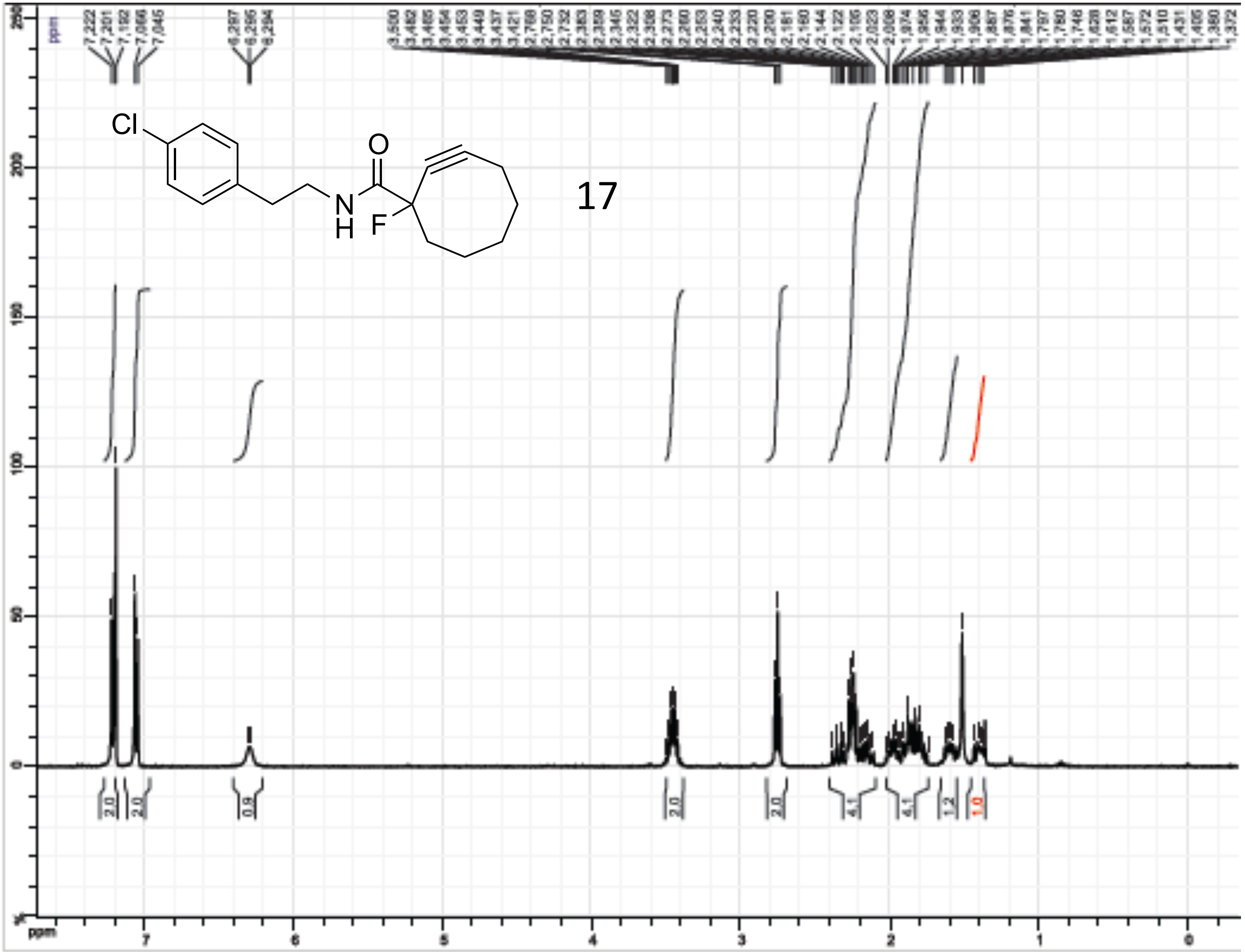
Cl
O
N
H
F
17
2.0
2.0
0.9
2.0
2.0
4.1
4.1
1.2
1.0
7
6
5
4
3
2
1
0
ppm

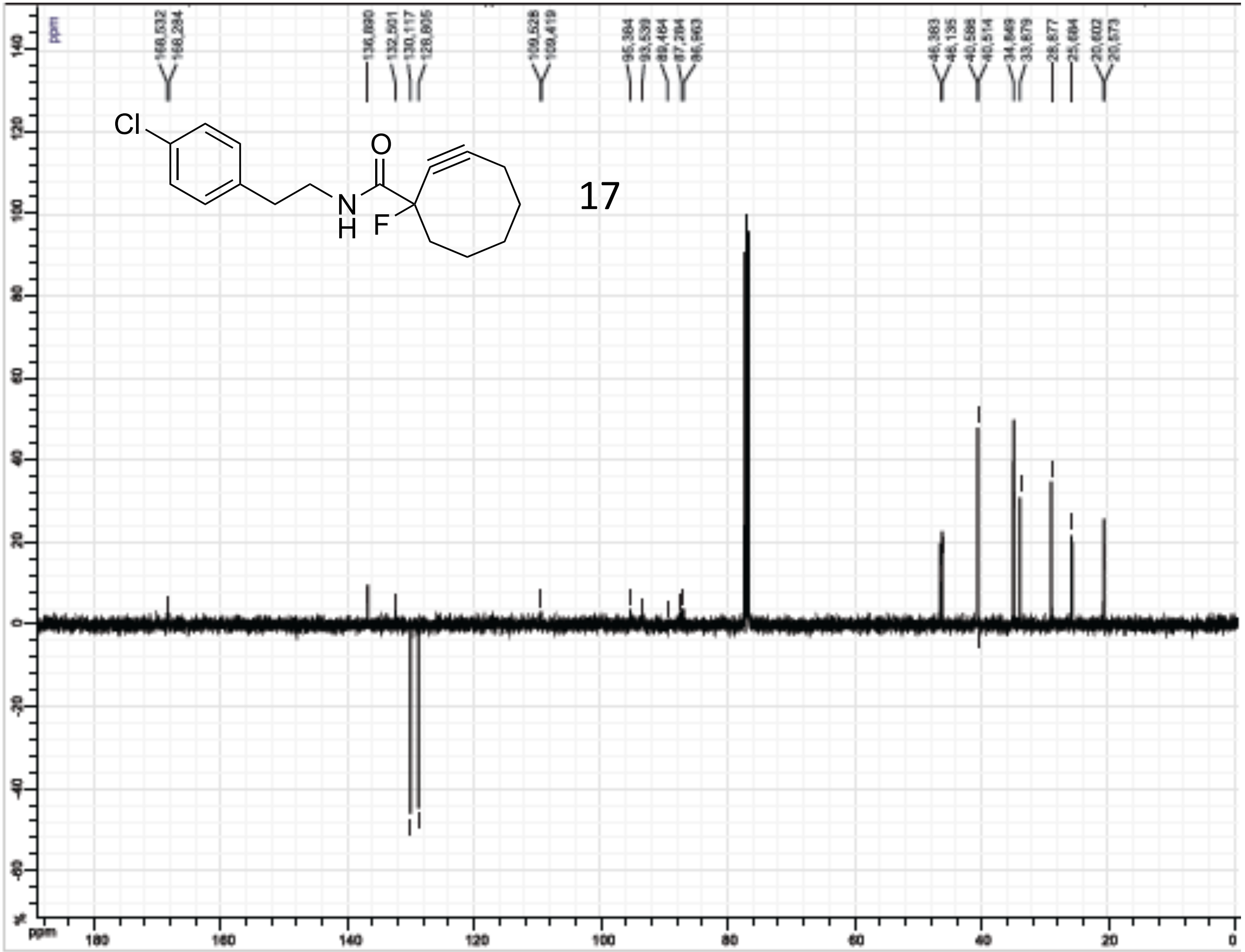
ppm
168.532
168.284
136.890
132.501
130.117
128.805
109.528
109.419
95.384
93.539
89.464
87.284
86.963
46.383
46.135
40.588
40.514
34.849
33.879
28.877
25.684
20.602
20.573
Cl
O
N
H
F
17

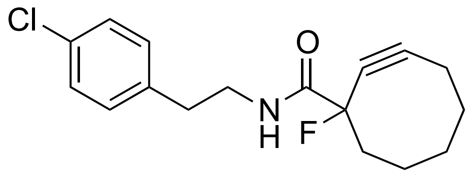
Cl
O
N
H
F

17

tr :
3.80

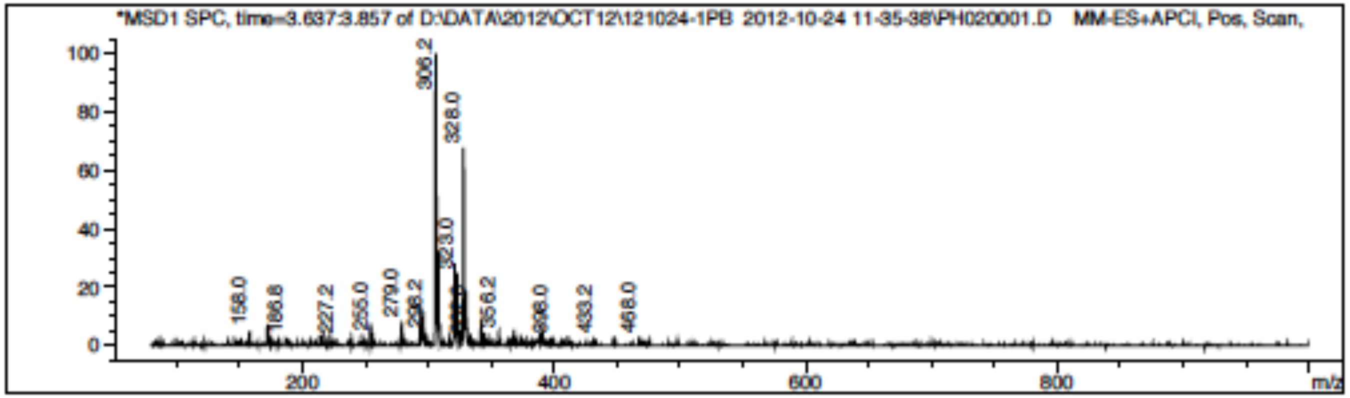
*MSD1 SPC, time=3.637:3.857 of D:\DATA\2012\OCT12\121024-1PB 2012-10-24 11-35-38\PH020001.D MM-ES+APCI, Pos, Scan,
306.2
328.0
323.0
158.0
186.8
227.2
255.0
279.0
298.2
356.2
398.0
433.2
468.0
m/z

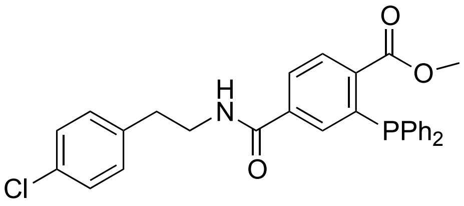
O
O
H
N
PPh2
Cl
O

18

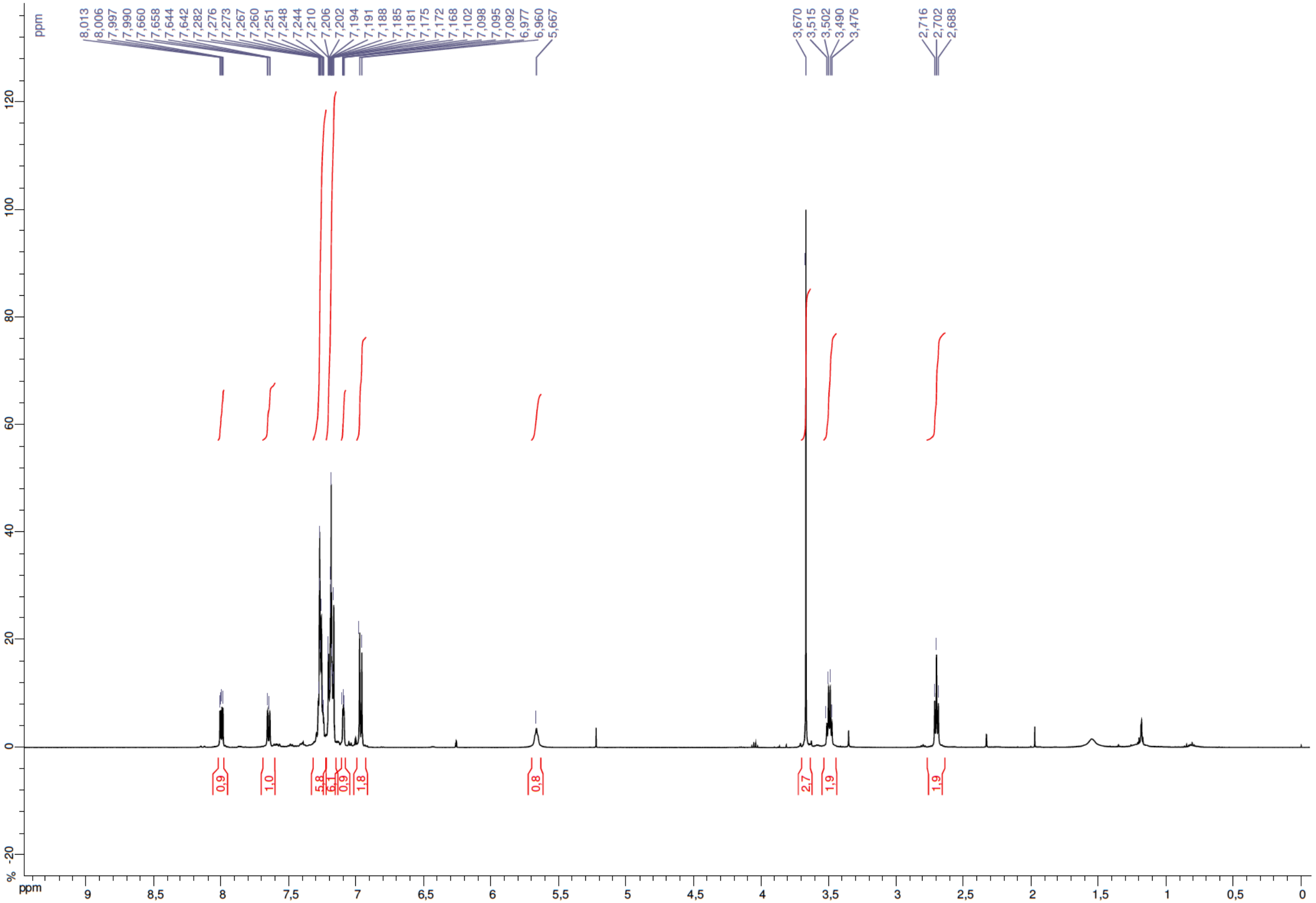
ppm
8,013
8,006
7,997
7,990
7,660
7,658
7,644
7,642
7,282
7,276
7,273
7,267
7,260
7,251
7,248
7,244
7,210
7,206
7,202
7,194
7,191
7,188
7,185
7,181
7,175
7,172
7,168
7,102
7,098
7,095
7,092
6,977
6,960
5,667
3,670
3,515
3,502
3,490
3,476
2,716
2,702
2,688
0,9
1,0
5,8
6,1
0,9
1,8
0,8
2,7
1,9
1,9
120
100
80
60
40
20
0
-20
%
ppm
9
8,5
8
7,5
7
6,5
6
5,5
5
4,5
4
3,5
3
2,5
2
1,5
1
0,5
0

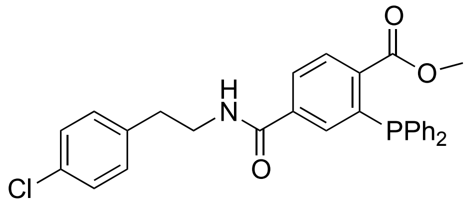
O
O
H
N
PPh2
O
Cl

18

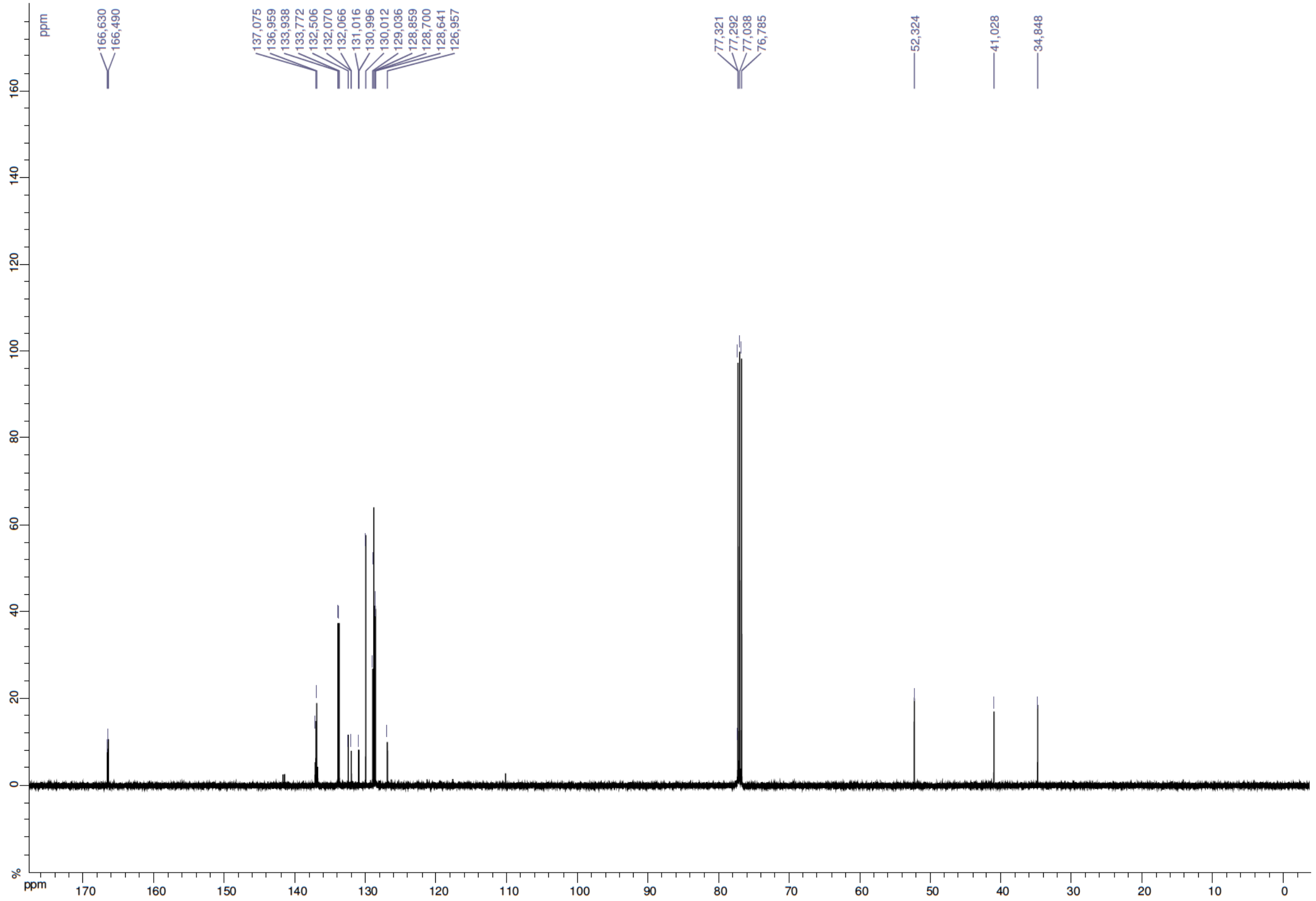
ppm
166,630
166,490
137,075
136,959
133,938
133,772
132,506
132,070
132,066
131,016
130,996
130,012
129,036
128,859
128,700
128,641
126,957
77,321
77,292
77,038
76,785
52,324
41,028
34,848
160
140
120
100
80
60
40
20
0
%
ppm
170
160
150
140
130
120
110
100
90
80
70
60
50
40
30
20
10
0

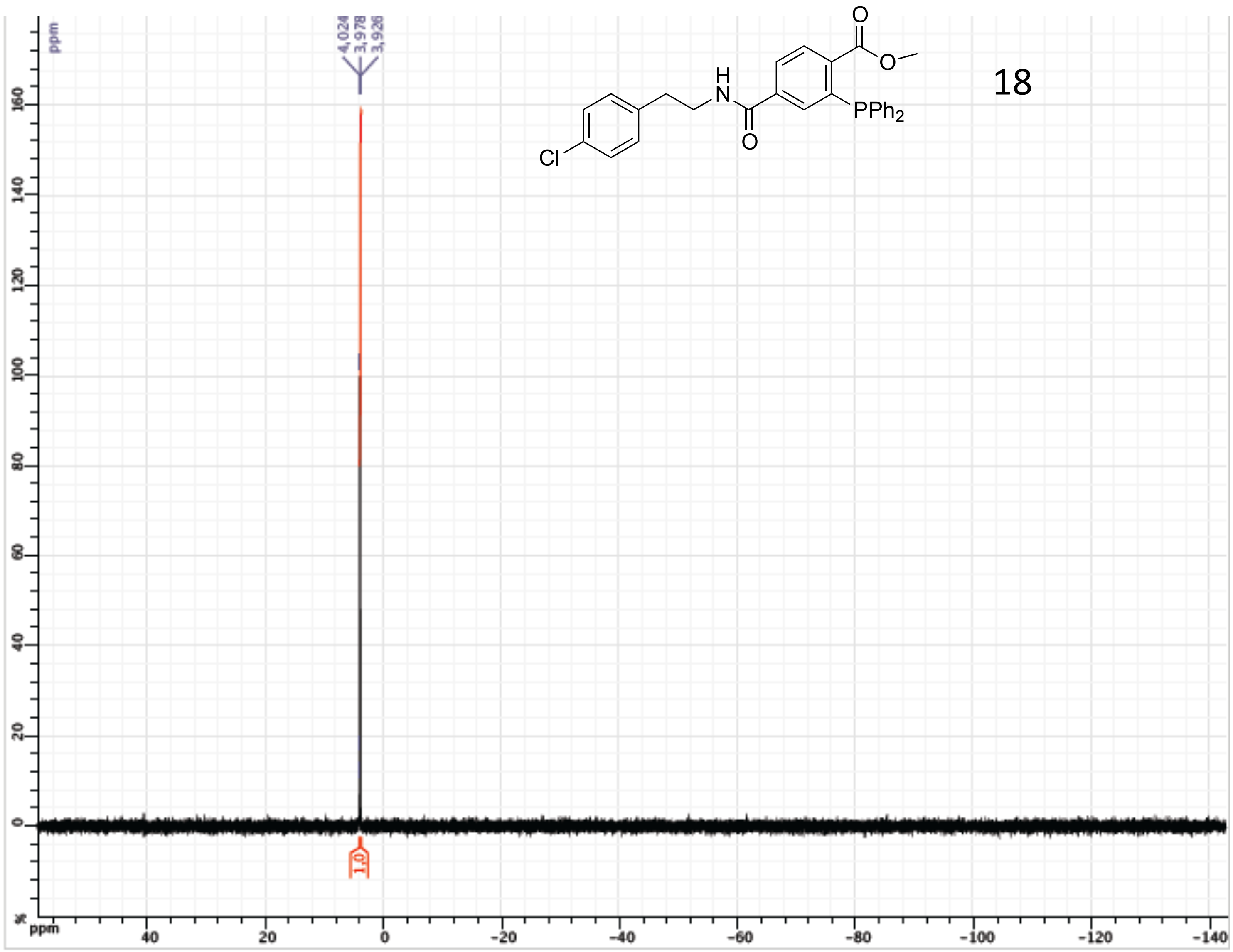

4,024
3,978
3,926
ppm
Cl
H
N
O
O
O
PPh2
1,0
ppm
40
20
0
-20
-40
-60
-80
-100
-120
-140

18

Spectrum Source	Fragmentor Voltage	Collision Energy	Ionization Mode
Peak (1) in "+ BPC(50.00000-1000.00000 [-39]) Scan"	120	0	ESI

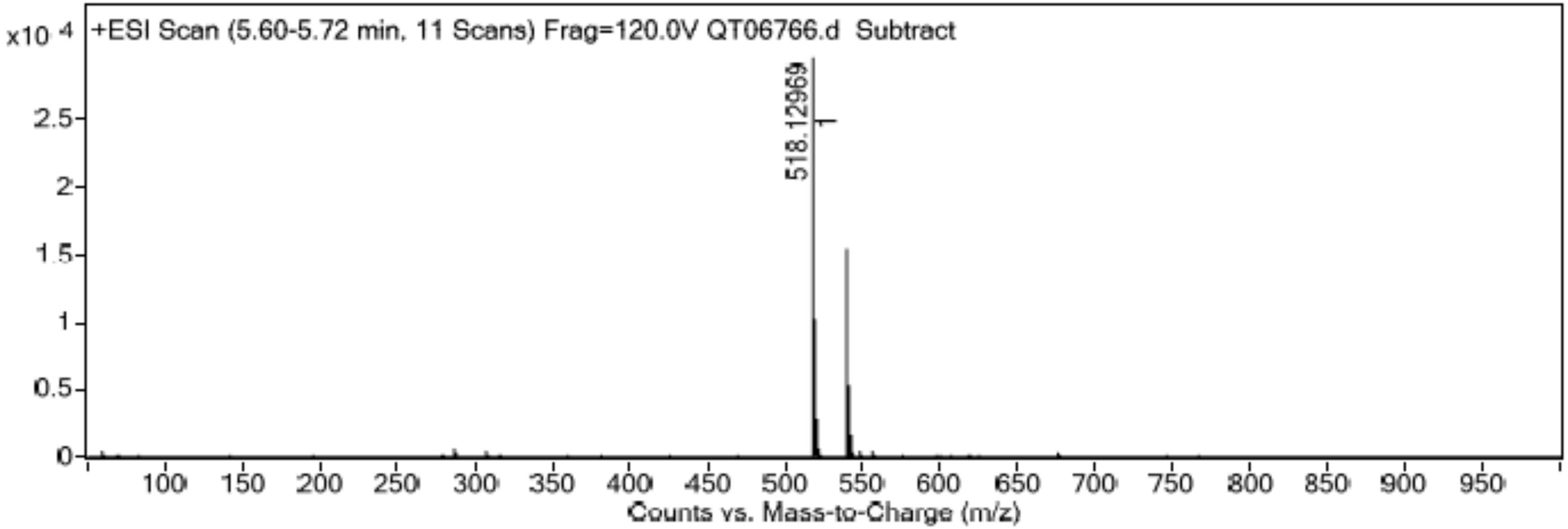

Peak List

m/z	z	Abund	Formula	Ion
518.12969	1	29659.2	C29 H26 Cl N O4 P	(M+H)+
519.13261	1	8903.9	C29 H26 Cl N O4 P	(M+H)+
520.1279	1	10246.6	C29 H26 Cl N O4 P	(M+H)+
540.11153	1	15458.7	C29 H25 Cl N Na O4 P	(M+Na)+
541.11442	1	4726.4	C29 H25 Cl N Na O4 P	(M+Na)+
542.10999	1	5406.5	C29 H25 Cl N Na O4 P	(M+Na)+
1057.23216	1	9573.5		
1058.23613	1	6382.2		
1059.23222	1	8363.2		
1060.23258	1	4690.1		

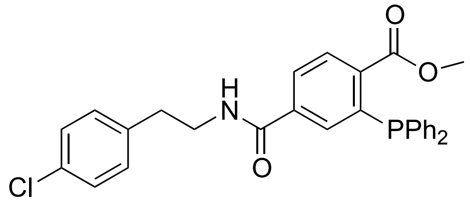

18

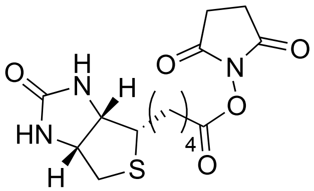

14

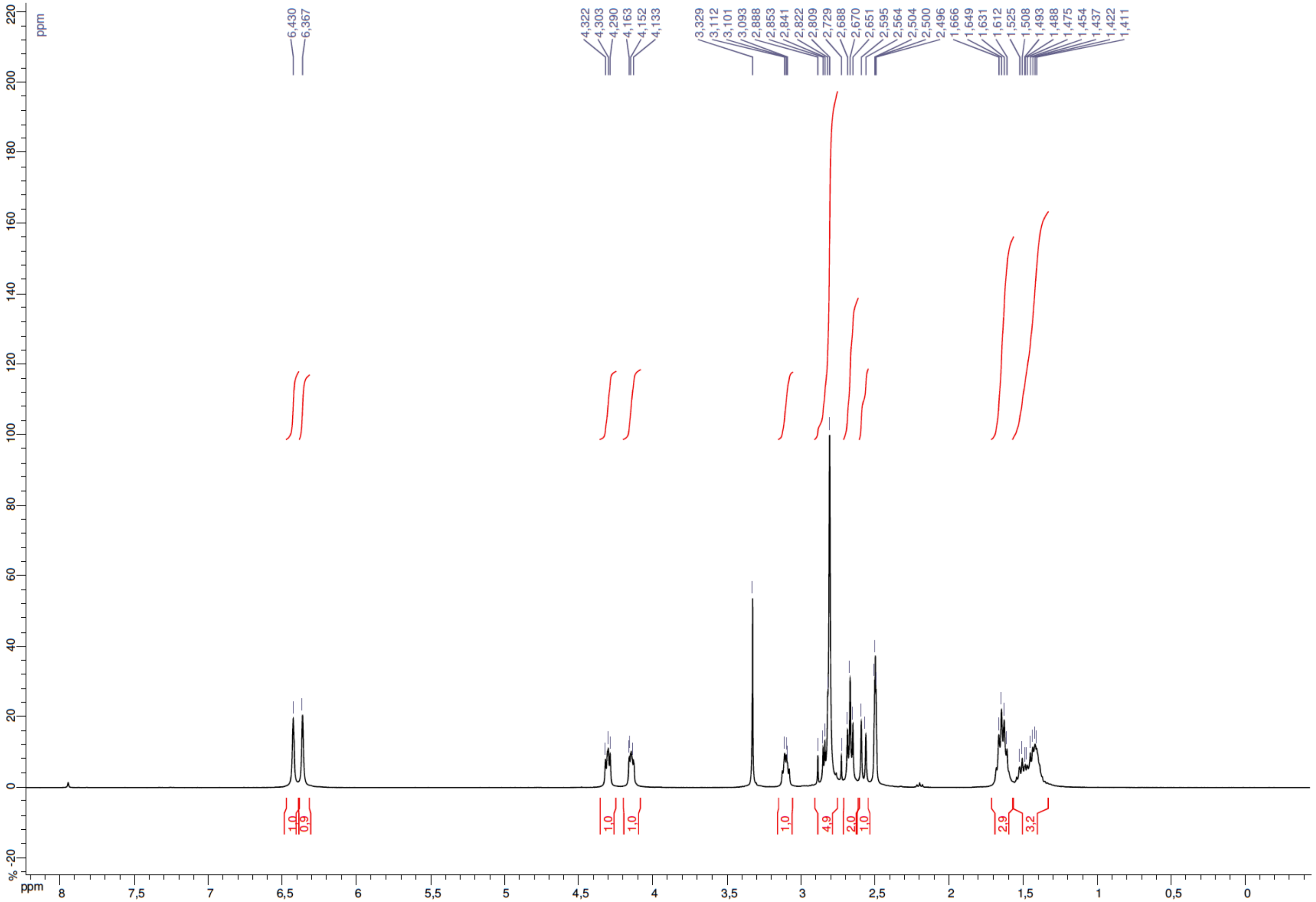

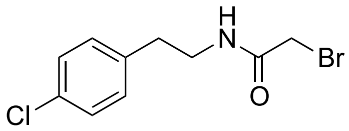
H
N
Br
O
Cl

DIB-5

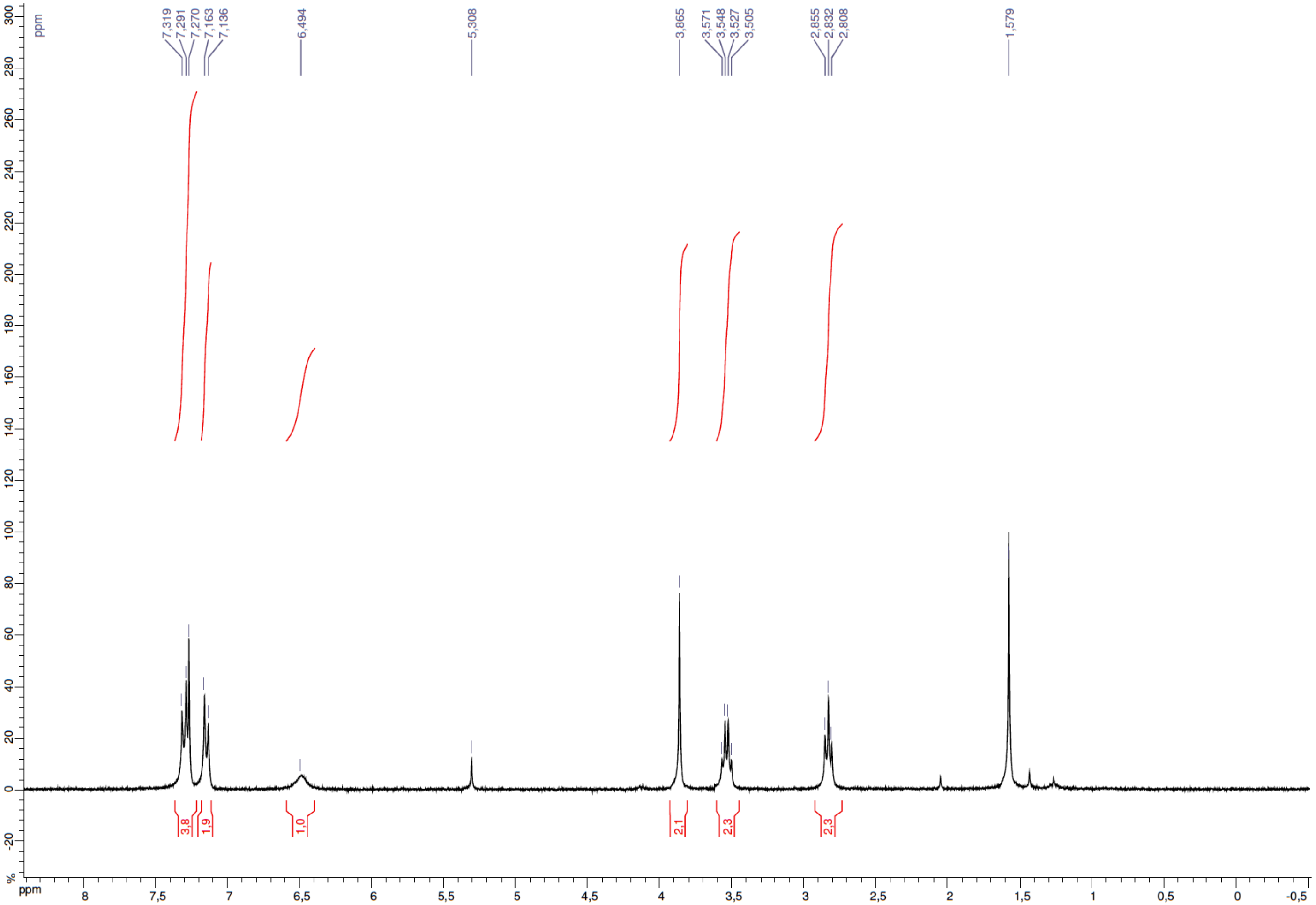
ppm
7,319
7,291
7,270
7,163
7,136
6,494
5,308
3,865
3,571
3,548
3,527
3,505
2,855
2,832
2,808
1,579
3,8
1,9
1,0
2,1
2,3
2,3
%
300
280
260
240
220
200
180
160
140
120
100
80
60
40
20
0
-20
ppm
8
7,5
7
6,5
6
5,5
5
4,5
4
3,5
3
2,5
2
1,5
1
0,5
0
-0,5

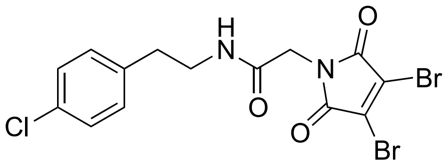
Cl
H
N
O
N
O
O
Br
Br

19

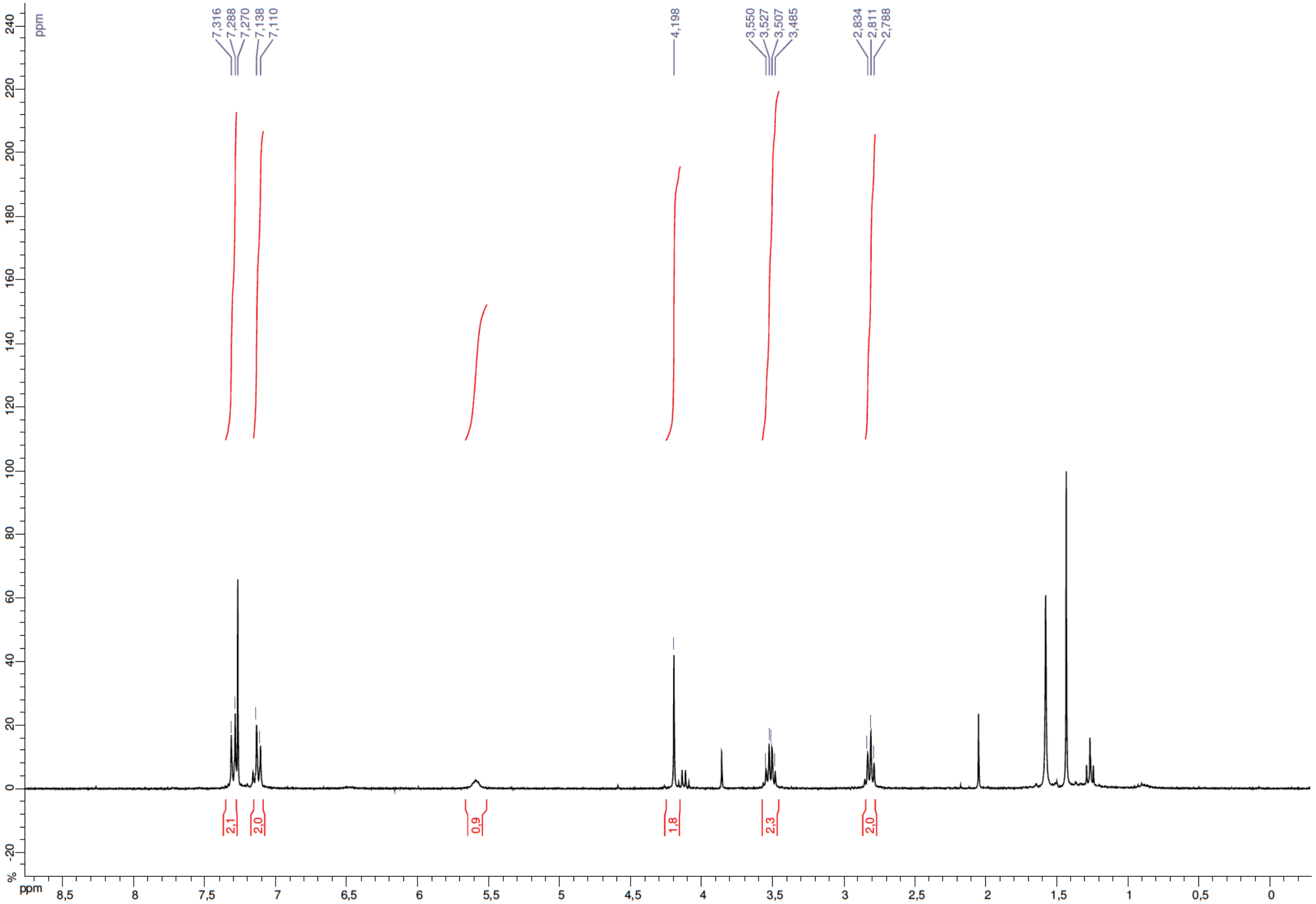
7,316
7,288
7,270
7,138
7,110
4,198
3,550
3,527
3,507
3,485
2,834
2,811
2,788
2,1
2,0
0,9
1,8
2,3
2,0
ppm
%
8,5
8
7,5
7
6,5
6
5,5
5
4,5
4
3,5
3
2,5
2
1,5
1
0,5
0

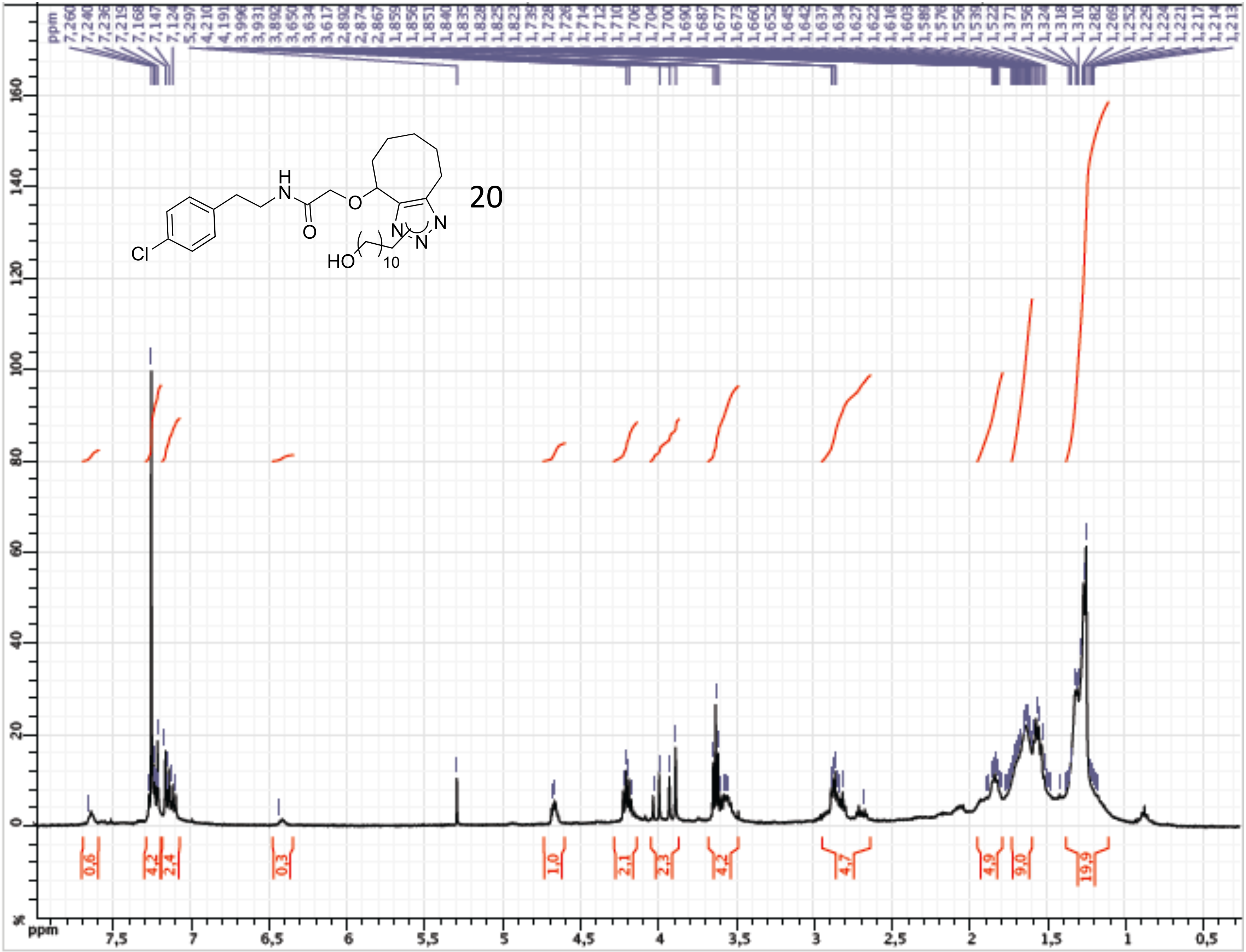
20
Cl
HO
10
ppm
0,6
4,2
2,4
0,3
1,0
2,1
2,3
4,2
4,7
4,9
9,0
19,9

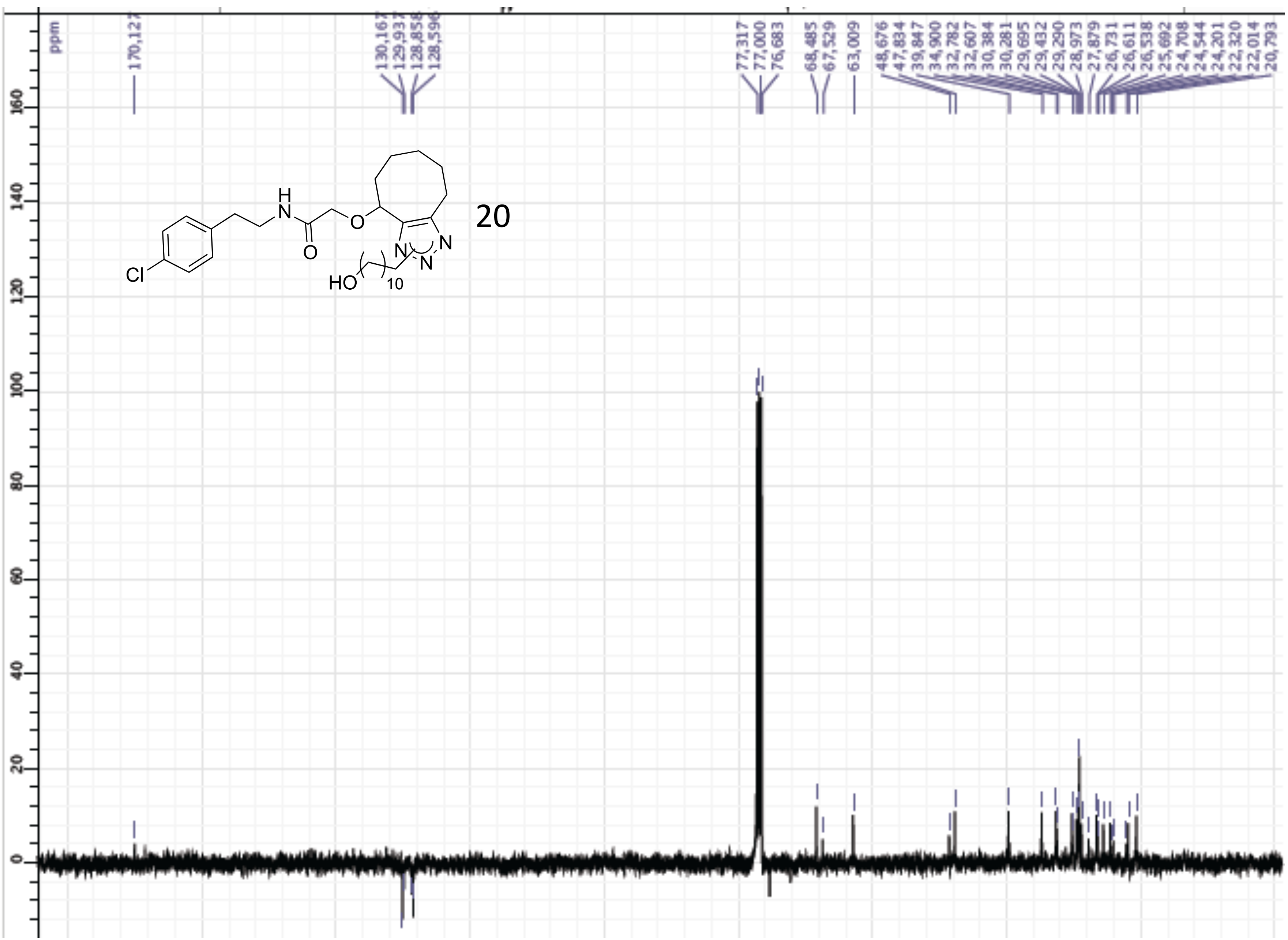
ppm
170,127
130,162
129,937
128,858
128,596
77,317
77,000
76,683
68,485
67,529
63,009
48,676
47,834
39,847
34,900
32,782
32,607
30,384
30,281
29,695
29,432
29,290
28,973
27,879
26,731
26,611
26,538
25,692
24,708
24,544
24,201
22,320
22,014
20,793
160
140
120
100
80
60
40
20
0
Cl
H
N
O
O
N
N
N
HO
10
20

Spectrum Source
Peak (3) in "+ BPC(50.00000-1000.00000 [-41])
Scan"

Fragmentor Voltage
120

Collision Energy
0

Ionization Mode
ESI

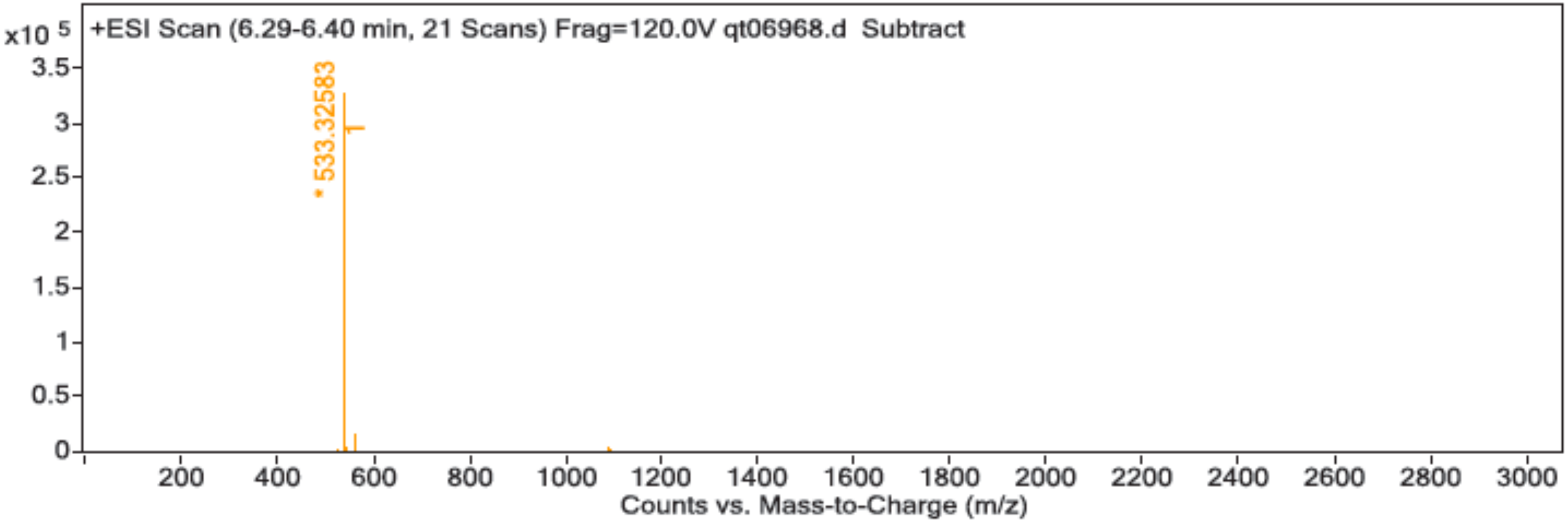

Peak List

m/z	z	Abund
533.32583	1	333150.7
534.32822	1	99100.5
535.32362	1	110958
536.32537	1	29148.3
555.30636		18801.2

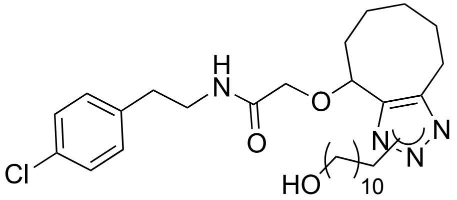

20

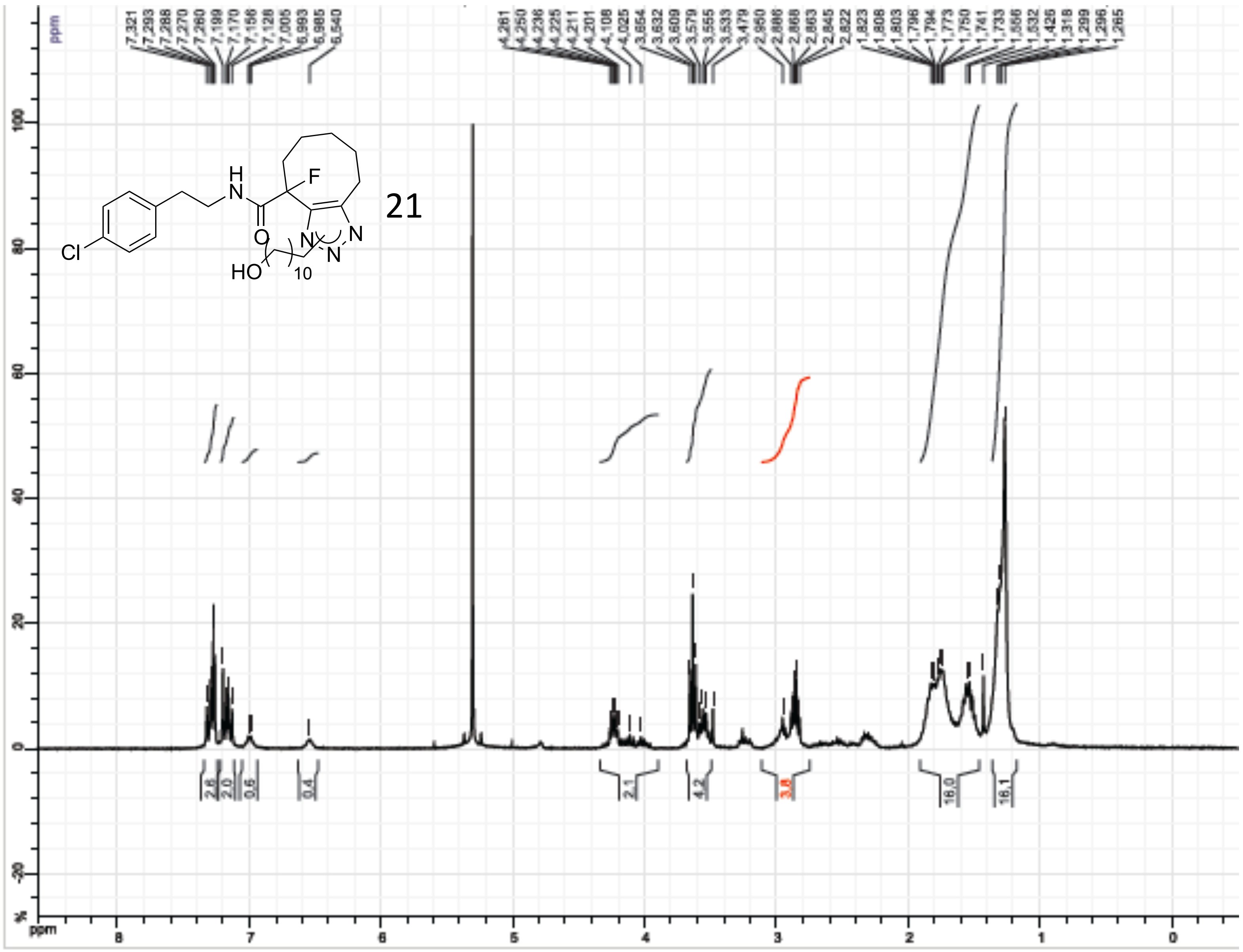

ppm
7,321
7,293
7,288
7,270
7,260
7,199
7,170
7,156
7,128
7,006
6,993
6,985
6,540
4,281
4,250
4,236
4,225
4,211
4,201
4,108
4,025
3,654
3,632
3,609
3,579
3,555
3,533
3,479
2,950
2,886
2,868
2,863
2,845
2,822
1,823
1,808
1,803
1,796
1,794
1,773
1,750
1,741
1,733
1,586
1,532
1,426
1,318
1,299
1,296
1,265
H
N
F
O
N
N
N
Cl
HO
10
21
2,6
2,0
0,6
0,4
2,1
4,2
3,8
18,0
16,1
%
ppm
8
7
6
5
4
3
2
1
0

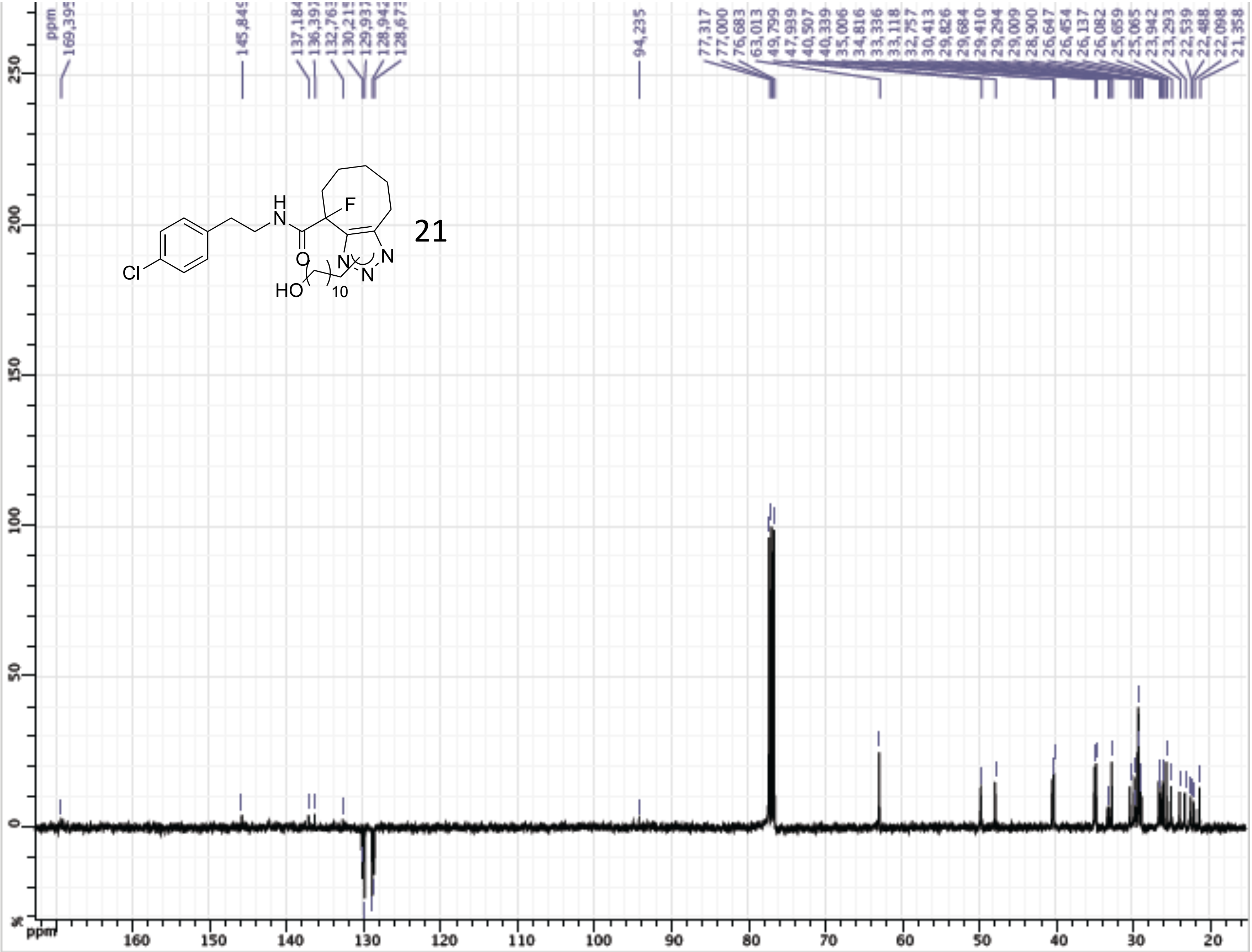

ppm
169,399
145,849
137,184
136,39
132,76
130,21
129,93
128,942
128,67
94,235
77,317
77,000
76,683
63,013
49,799
47,939
40,507
40,339
35,006
34,816
33,336
33,118
32,757
30,413
29,826
29,684
29,410
29,294
29,009
28,900
26,647
26,454
26,137
26,082
25,659
25,065
23,942
23,293
22,539
22,488
22,098
21,358
Cl
H
N
O
F
N
N
N
HO
10
21
250
200
150
100
50
0
160
150
140
130
120
110
100
90
80
70
60
50
40
30
20

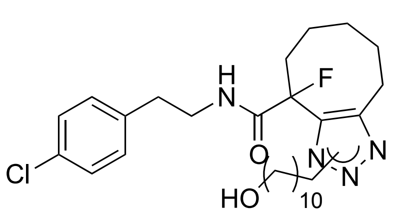

21

tr :
4.94

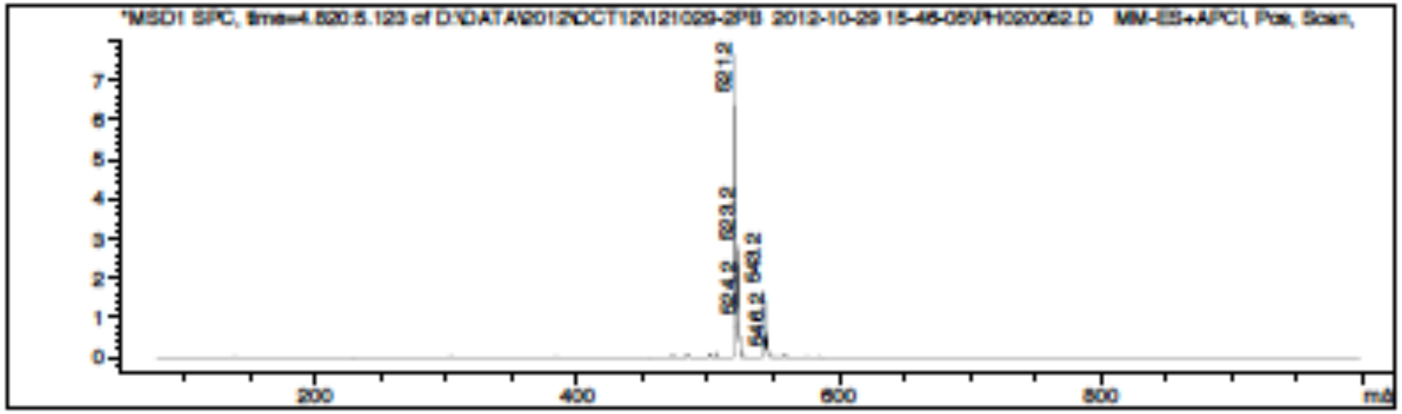

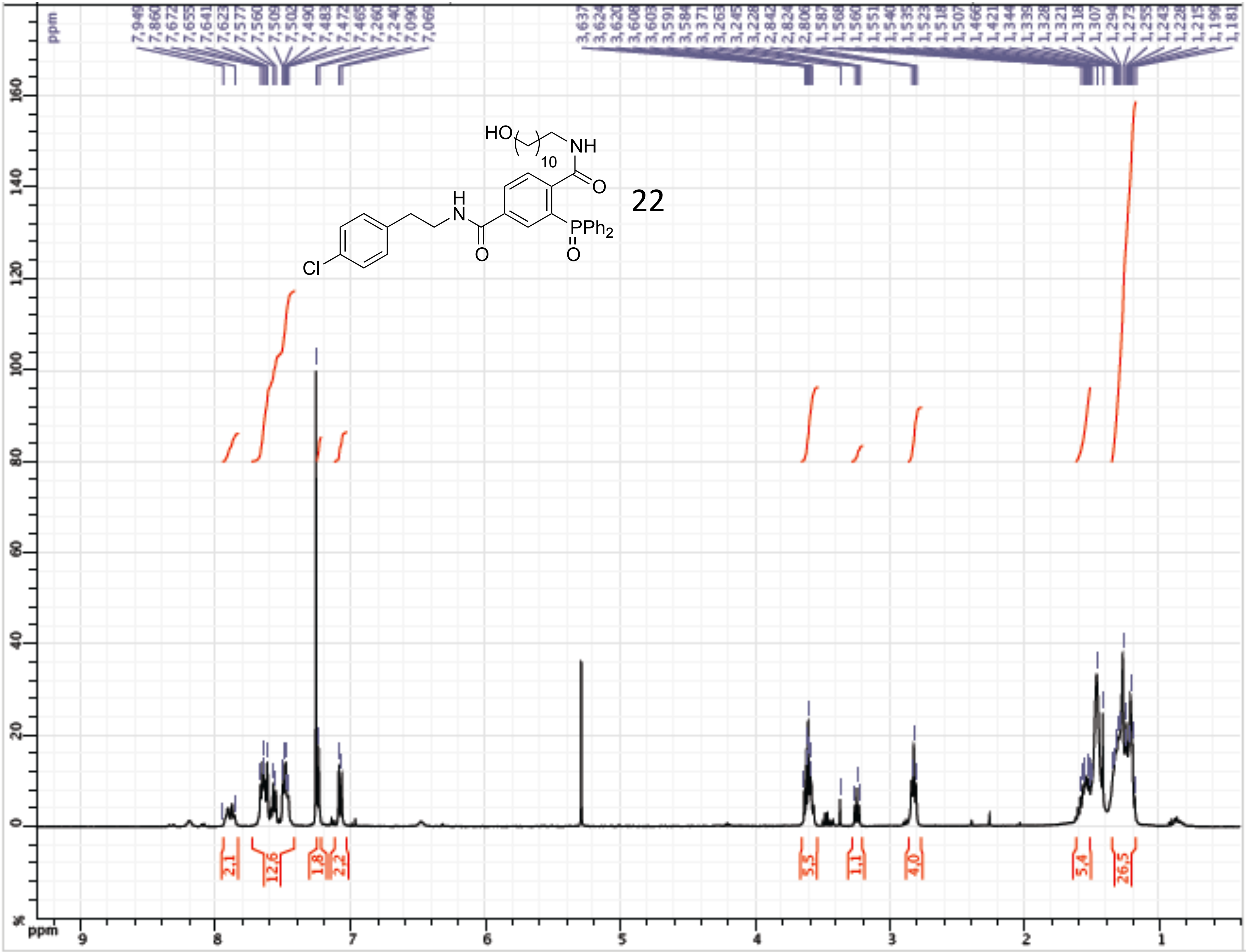
22
HO
NH
10
O
H
N
PPh2
O
O
Cl
ppm
2,1
12,6
1,8
2,2
5,5
1,1
4,0
5,4
26,5

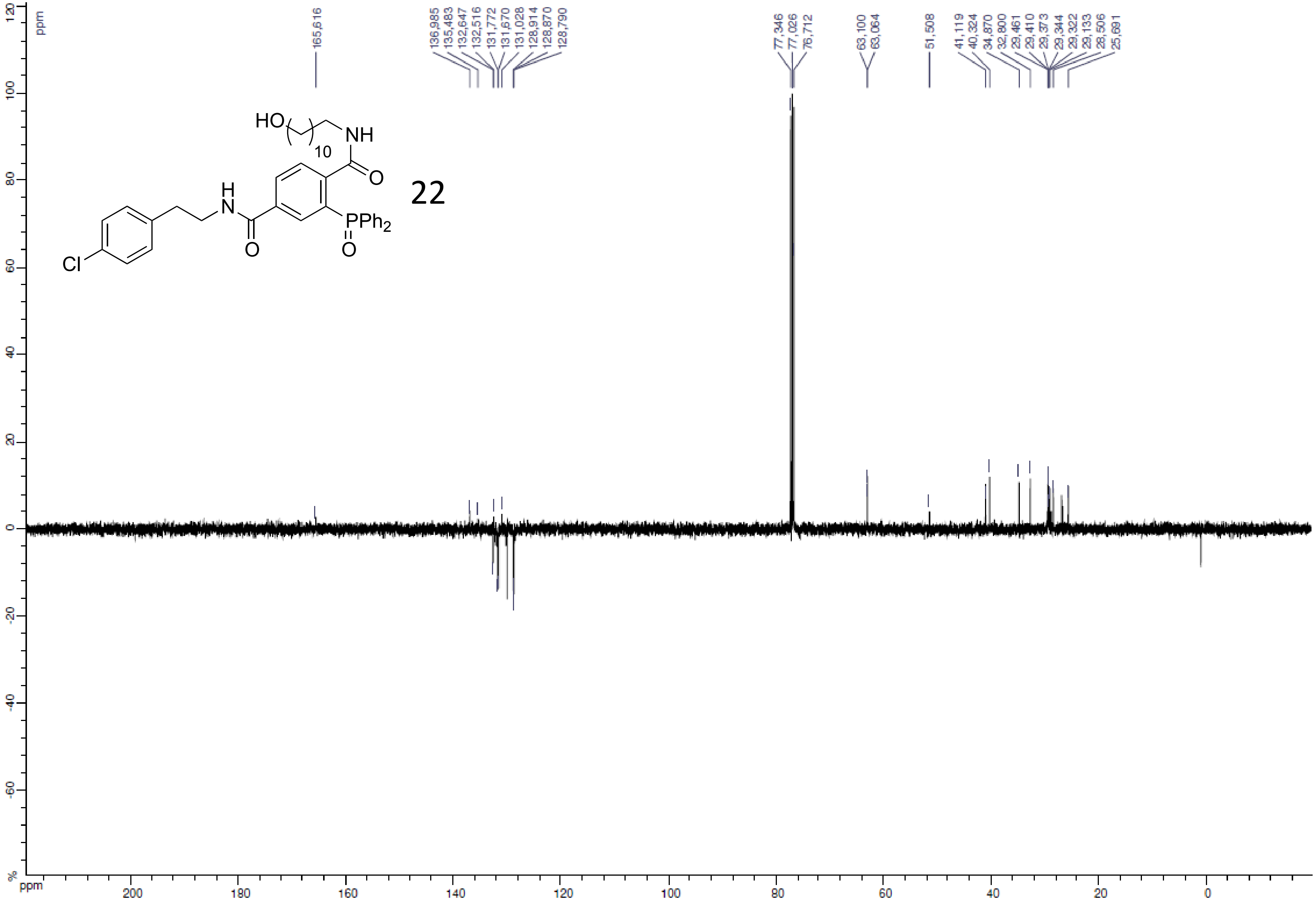

165,616
136,985
135,483
132,647
132,516
131,772
131,670
131,028
128,914
128,870
128,790
77,346
77,026
76,712
63,100
63,064
51,508
41,119
40,324
34,870
32,800
29,461
29,410
29,373
29,344
29,322
29,133
28,506
25,691
HO
NH
10
O
H
N
O
PPh2
O
Cl
22
ppm
%
200
180
160
140
120
100
80
60
40
20
0

Spectrum Source	Fragmentor Voltage	Collision Energy	Ionization Mode
Peak (1) in "+ BPC(50.00000-1000.00000 [-39]) Scan"	120	0	ESI

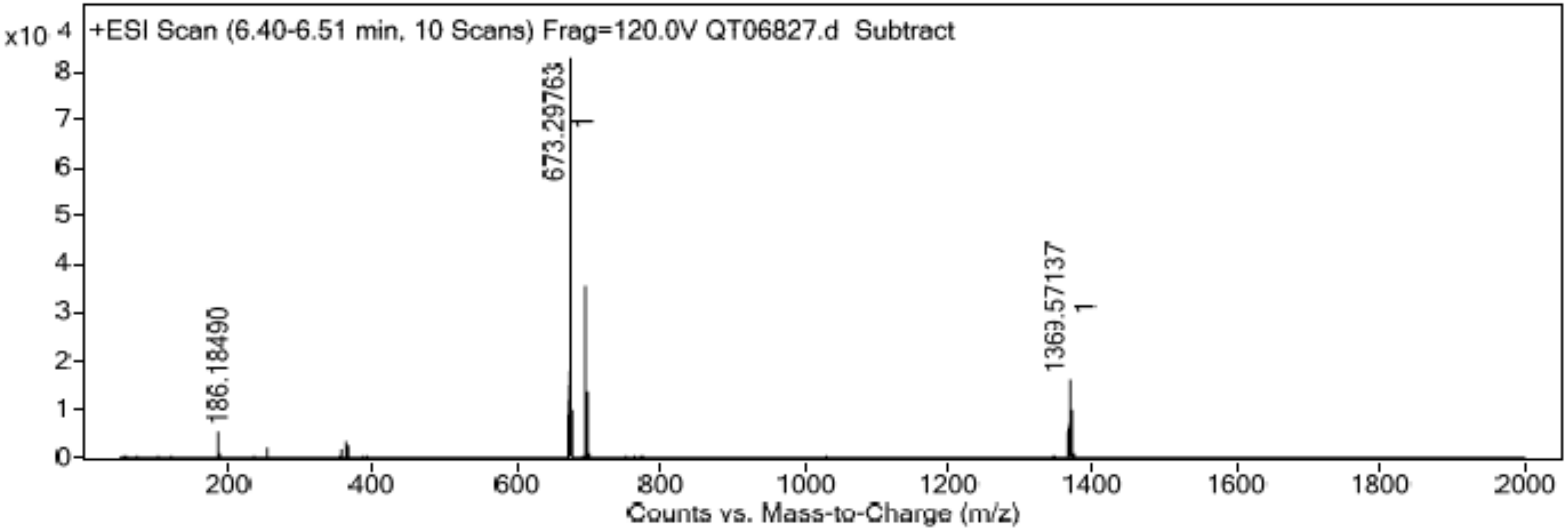

Peak List

m/z	z	Abund	Formula	Ion
673.29763	1	82929.9	C39 H47 Cl N2 O4 P	(M+H)+
674.30052	1	32398.7	C39 H47 Cl N2 O4 P	(M+H)+
675.29632	1	30486.7	C39 H47 Cl N2 O4 P	(M+H)+
676.29824	1	10265.1	C39 H47 Cl N2 O4 P	(M+H)+
695.27947	1	35897.2	C39 H46 Cl N2 Na O4 P	(M+Na)+
696.28238	1	14262.2	C39 H46 Cl N2 Na O4 P	(M+Na)+
697.27873	1	13713.6	C39 H46 Cl N2 Na O4 P	(M+Na)+
1367.57042	1	15316.3		
1368.57333	1	12999.6		
1369.57137	1	16093.8		

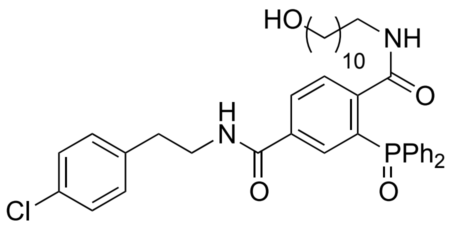

22

Spectrum Source	Fragmentor Voltage	Collision Energy	Ionization Mode
Peak (6) in "+ BPC(50.00000-1000.00000 [-67]) Scan Sub"	120	0	ESI

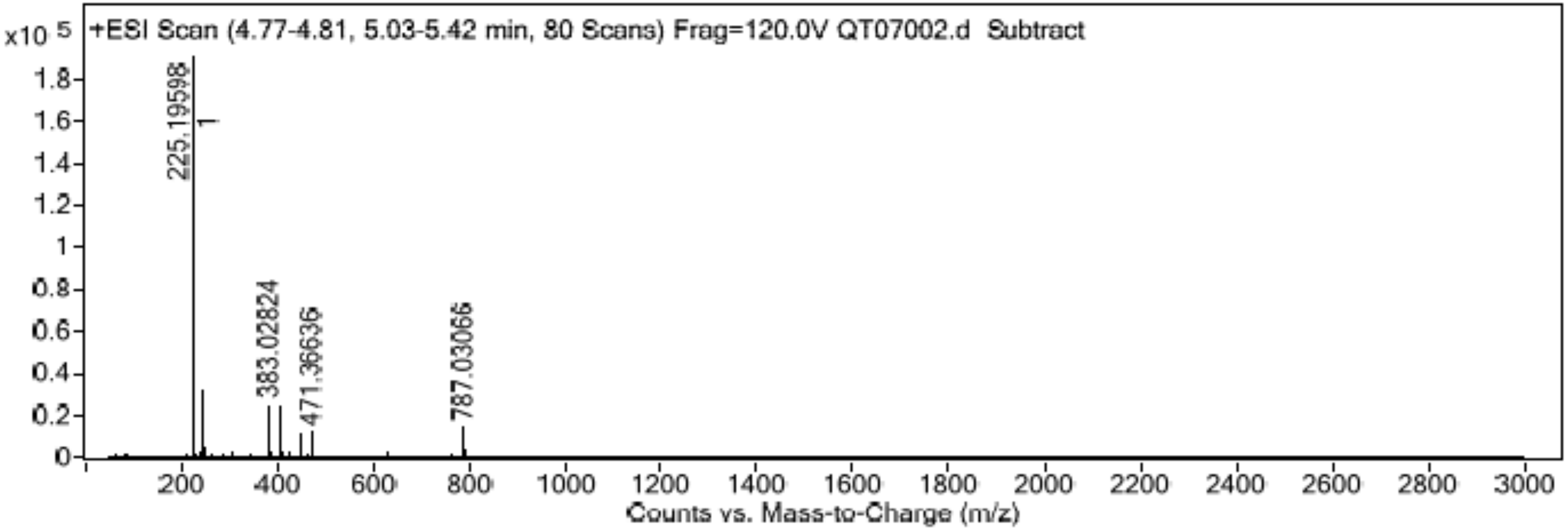

Peak List

m/z	z	Abund	Formula	Ion
225.19598	1	190704.2	C13 H25 N2 O	(M+H)+
226.19867	1	24105.6	C13 H25 N2 O	(M+H)+
247.1775		32058.2		
383.02824		25714.4	C16 H16 Cl N2 O3 S2	(M+H)+
405.01007		24539.8		
407.01106		10595.2		
449.38429		11881.5		
471.36636		12085.6		
787.03066		14435.7		
789.02881		13218.8		

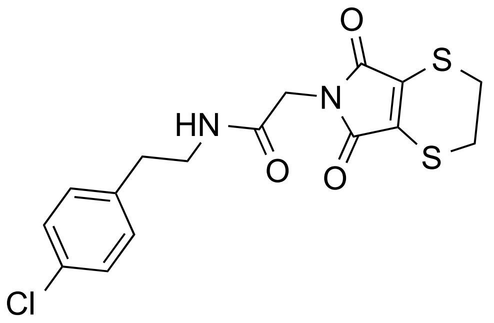

23
